# Supplementary material for: Synthesis, Characterization and Biological Evaluation of Benzothiazole–Isoquinoline Derivative
Source: Molecules. 2022 Dec 19;27(24):9062. doi: 10.3390/molecules27249062 (PMC9782539; doi:10.3390/molecules27249062)
Supplement: Supplementary file 1 [file molecules-27-09062-s001.zip › SI.pdf]

Supplementary Materials

Synthesis and Biological Activity Evaluation of Benzothiazole-isoquinoline Derivatives

Wei-Hua Liu[1,†], Zhi-Wen He[1,†], Dong-Hai Zhao[2], Yi-Ming Hu[1], Yu-Xia Zhu[1], Ling-Jian Zhang[1], Lian-Hai Jin[2], Li-Ping Guan[1,*], Si-Hong Wang[3,*]

[1] Food and Pharmacy College, Zhejiang Ocean University, Zhejiang, Zhoushan 316022, P. R. China

[2] Pharmacy College, Jilin Medical University, Jilin, Jilin 132013, P. R. China

[3] Key Laboratory of Natural Resource of the Changbai Mountain and Functiaonal Molecules, Ministry of Education, Yanbian University, Jilin, Yanji 133000, P. R. China

[†] These authors contributed equally to this work.

[*] Corresponding author.
E-mail address: glp730@163.com (L.P. Guan); shwang@ybu.edu.cn (S.-H. Wang).

Table of Content

1. AO Fluorescent Staining Analysis Derivatives (**4b-4d**, **4f**,**4j**,**4i**)

2. HPLC Data of Derivatives (**4a-4p**)

3. NMR Data of Derivatives (**4a-4p**)

1. AO Fluorescent Staining Analysis of Derivatives (**4b-4d**, **4f**,**4j**,**4i**)

10X 20X

control：

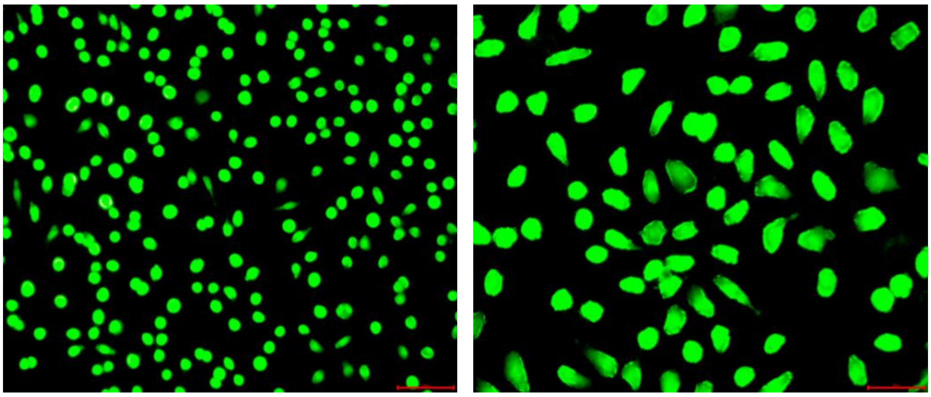

4b:

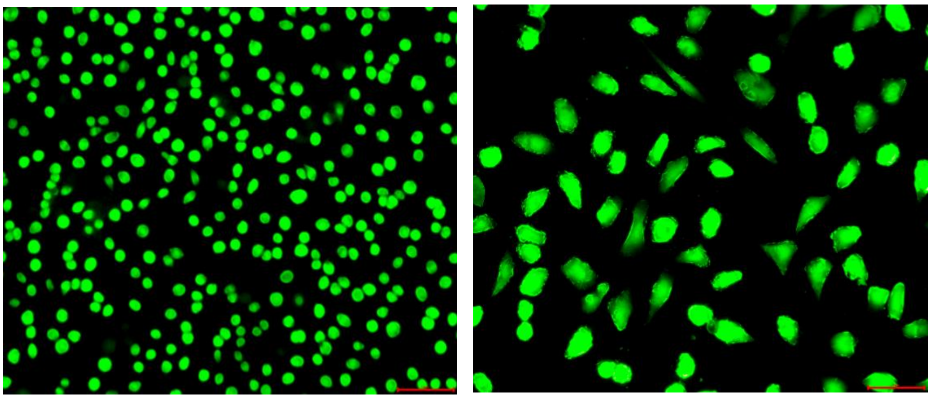

4c:

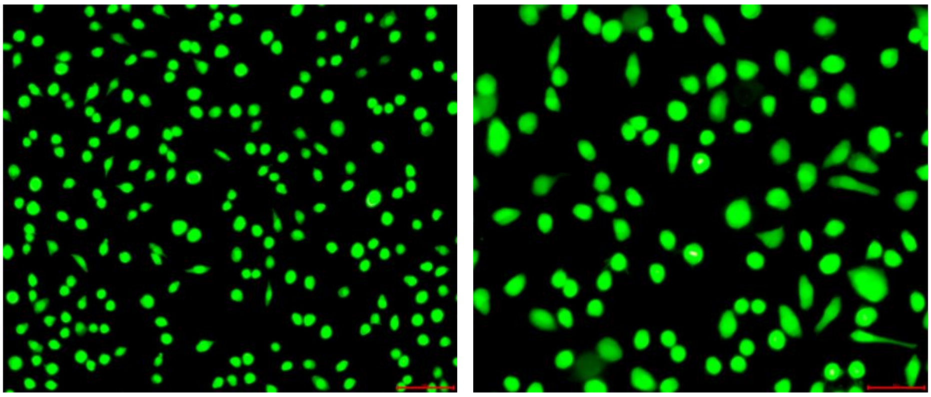

4d:

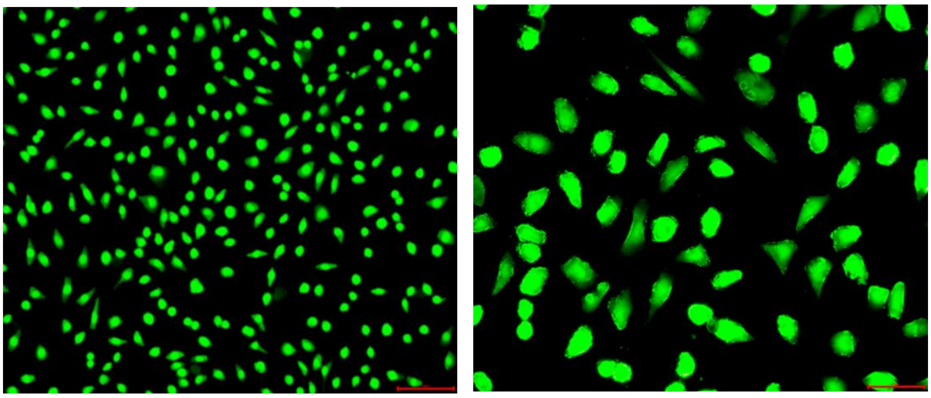

4f:

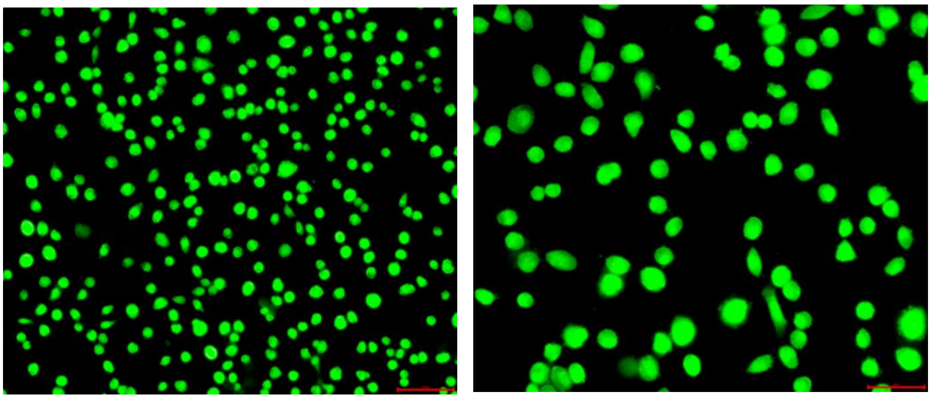

4g:

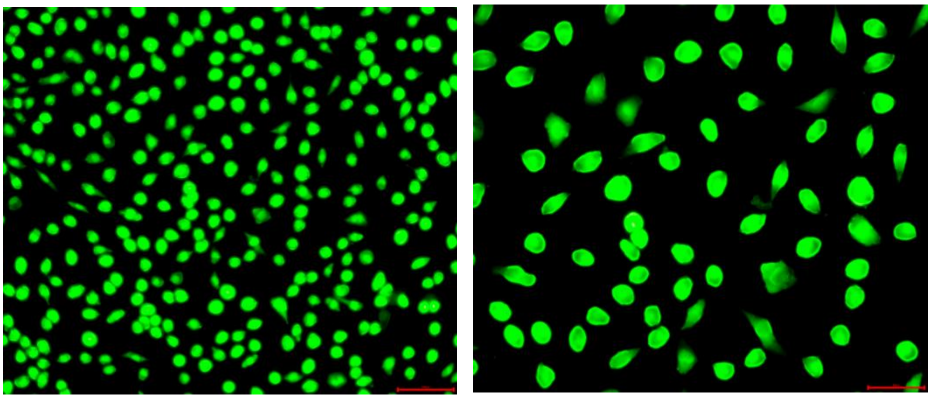

4i:

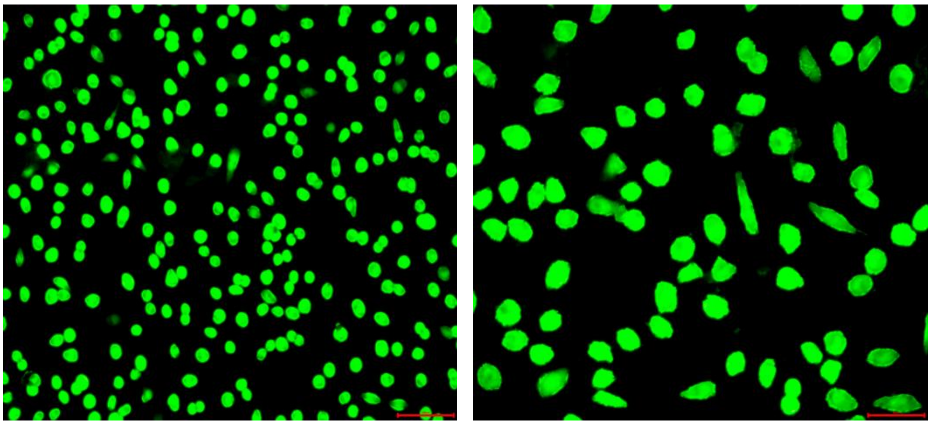

Figure S1. *The effect of benzothiazole-isoquinoline derivatives (**4b-4d**, **4f**, **4g**, **4i**) on the proliferation of L929 cells*

2. HPLC Data of Derivatives (**4a-4p**)

4a:

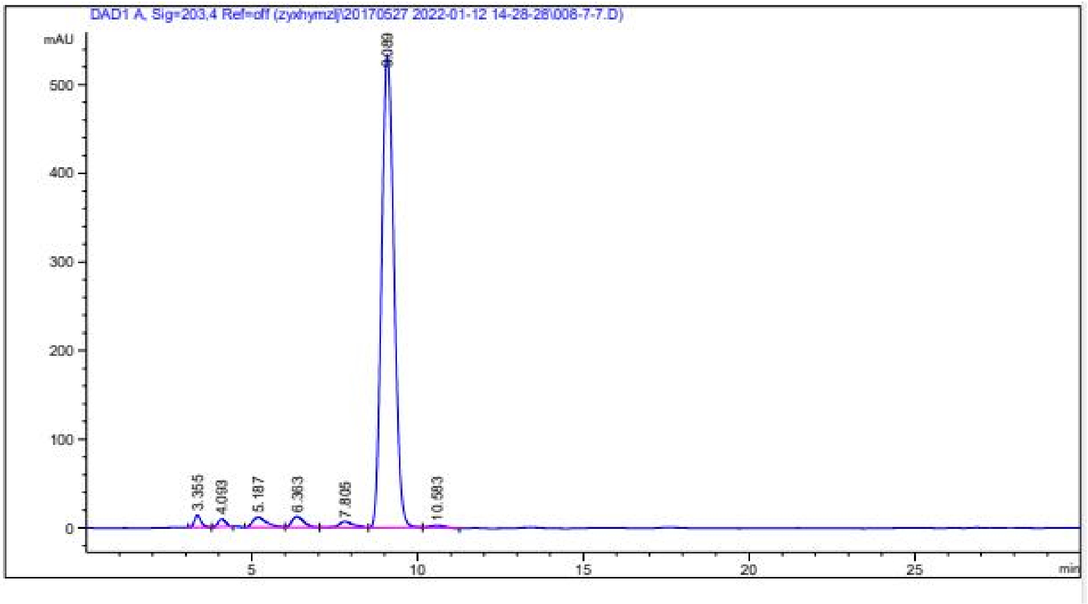

Figure S2. *The HPLC data of* ***4a***

4b

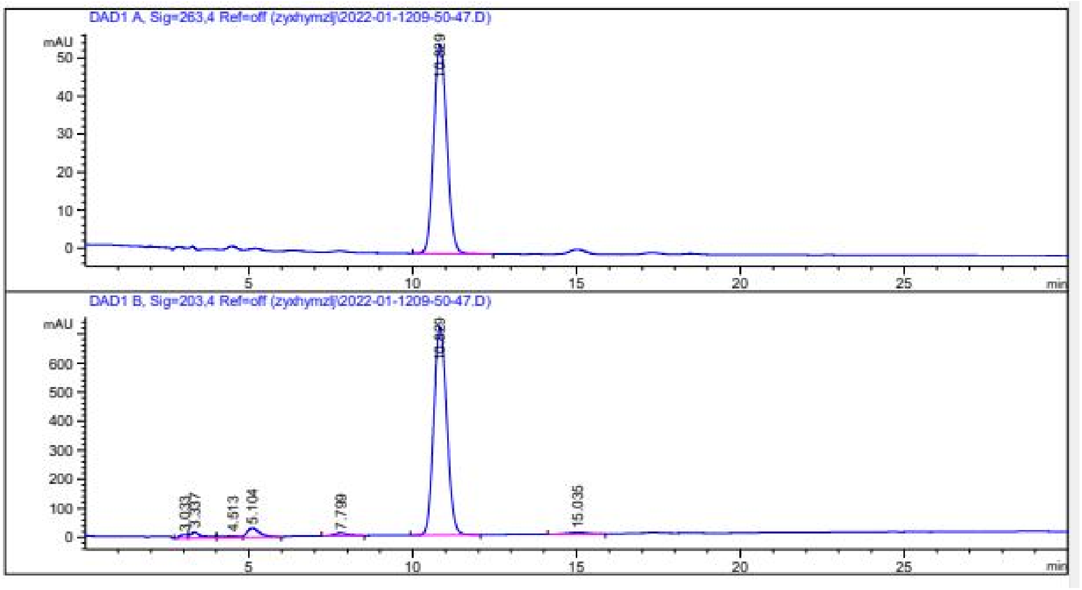

Figure S3. *The HPLC data of* ***4b***

4c:

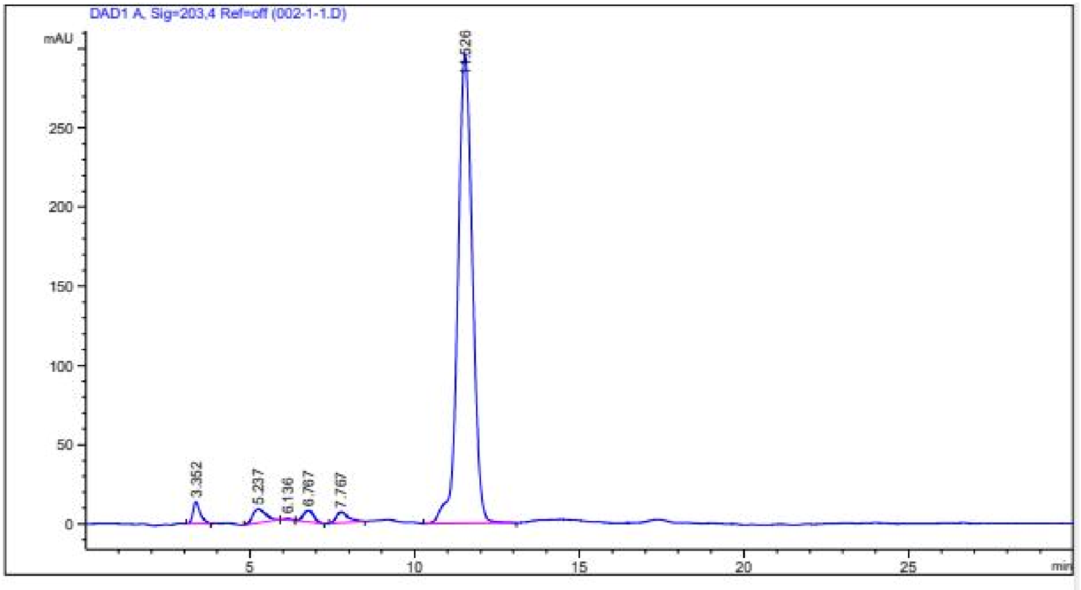

Figure S4. *The HPLC data of* ***4c***

4d:

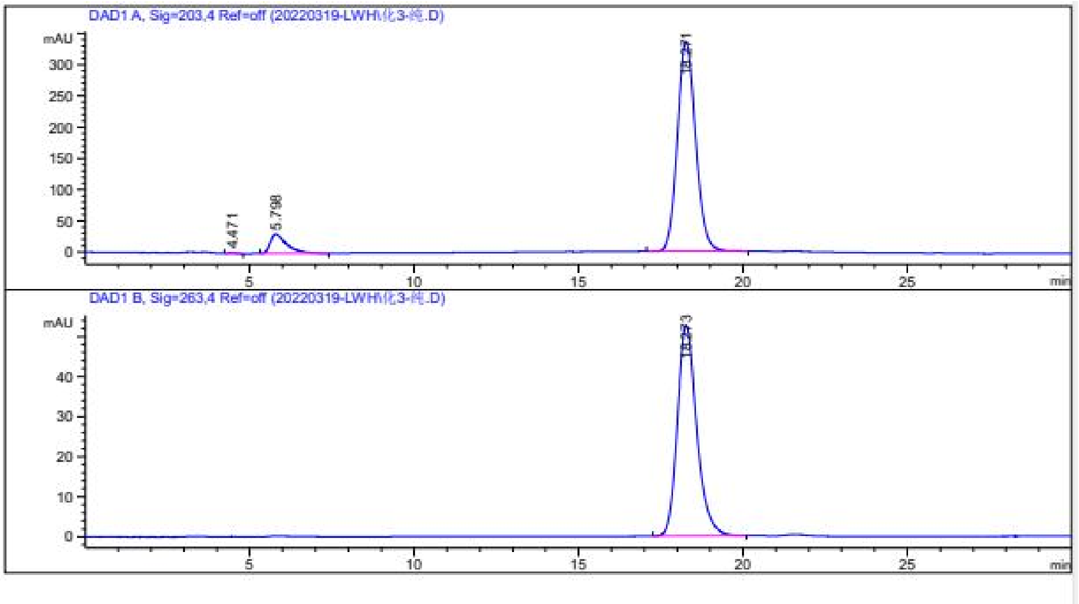

Figure S5. *The HPLC data of* ***4d***

4e:

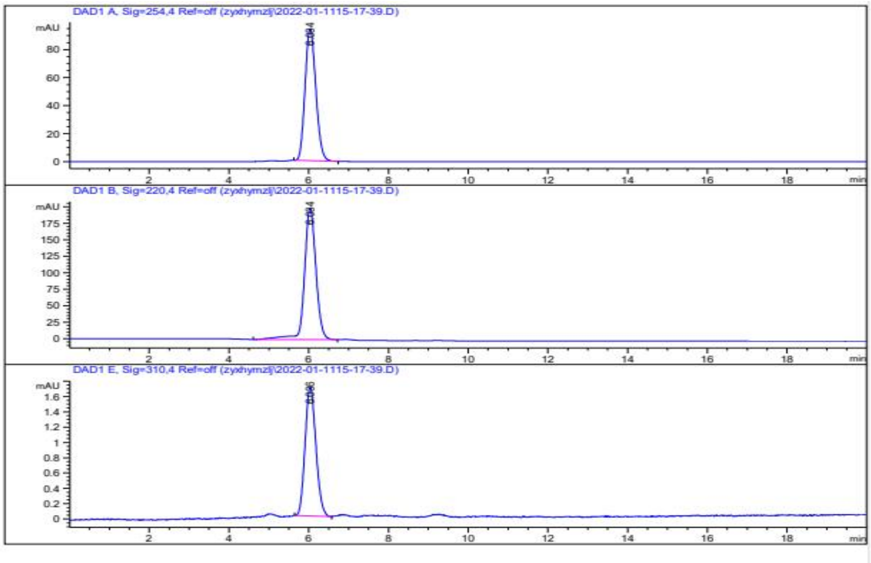

Figure S6. *The HPLC data of* ***4e***

4f:

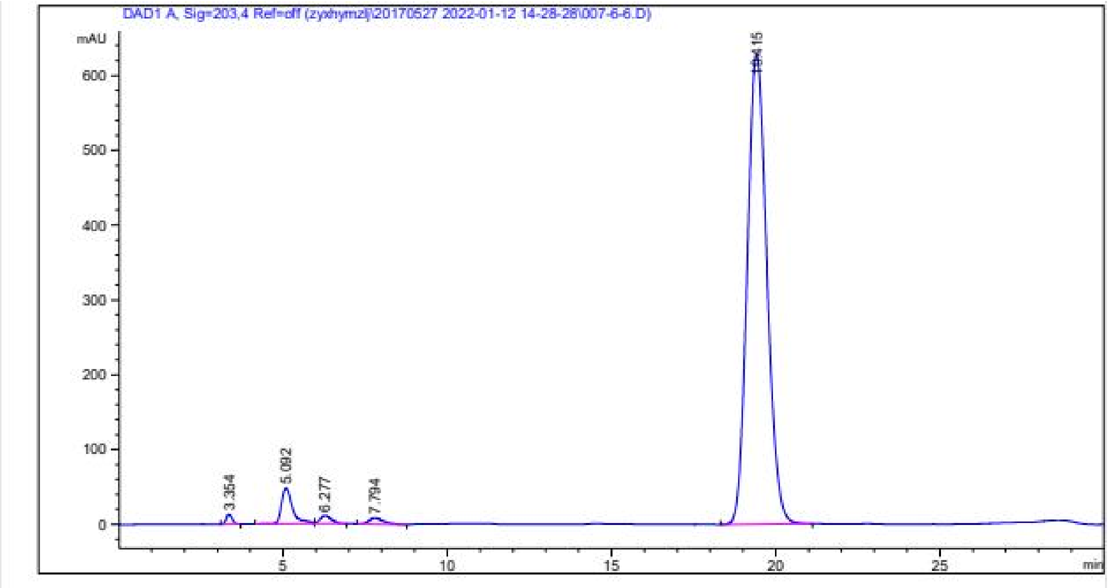

Figure S7. *The HPLC data of* ***4f***

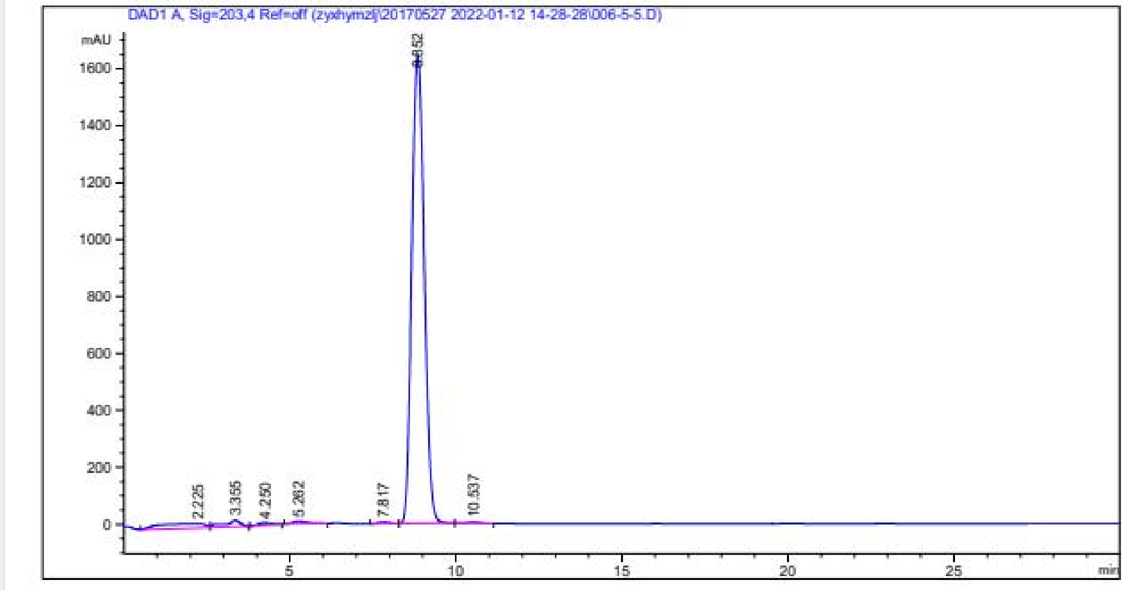

Figure S8. *The HPLC data of **4g***

4h:

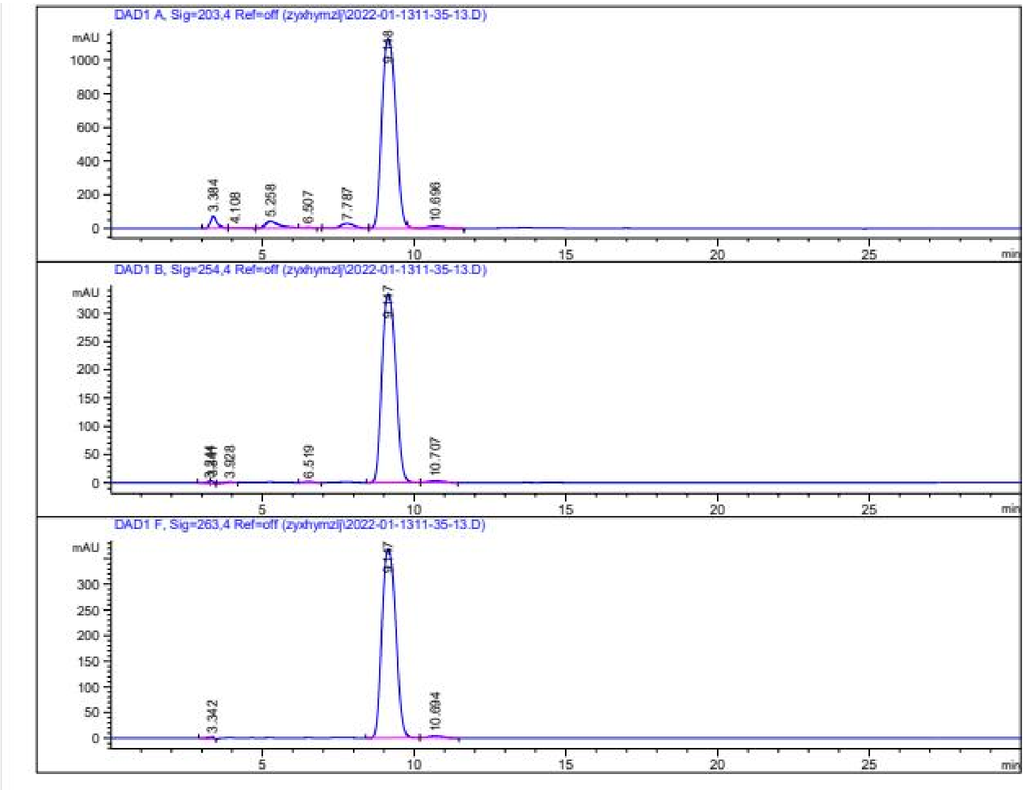

Figure S9. *The HPLC data of **4h***

4i:

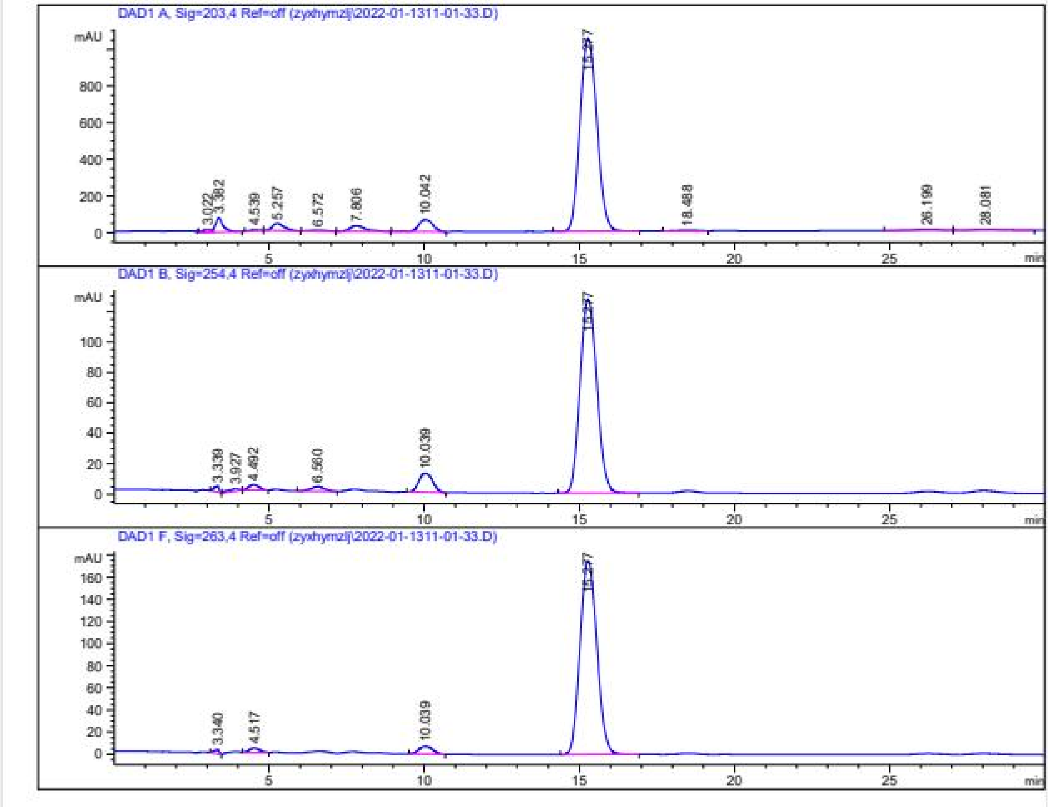

Figure S10. *The HPLC data of* ***4i***

4j:

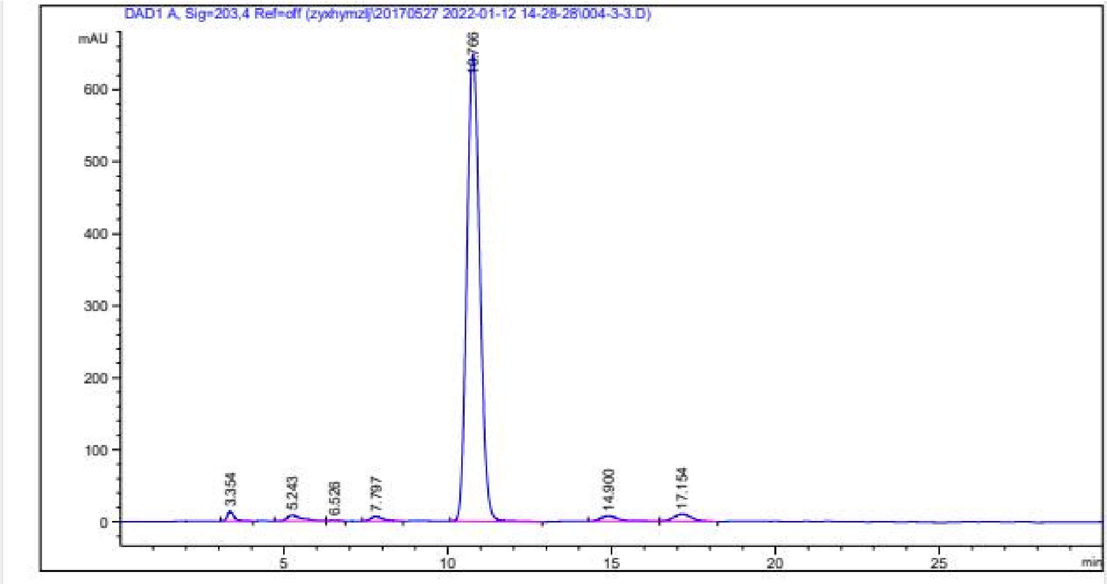

Figure S11. *The HPLC data of* ***4k***

4k:

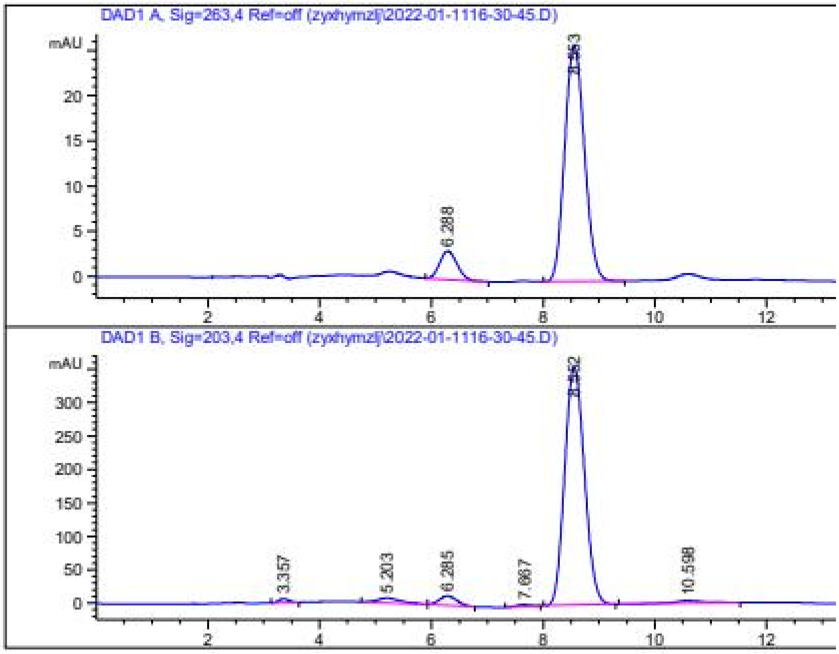

Figure S12. *The HPLC data of* ***4k***

4l:

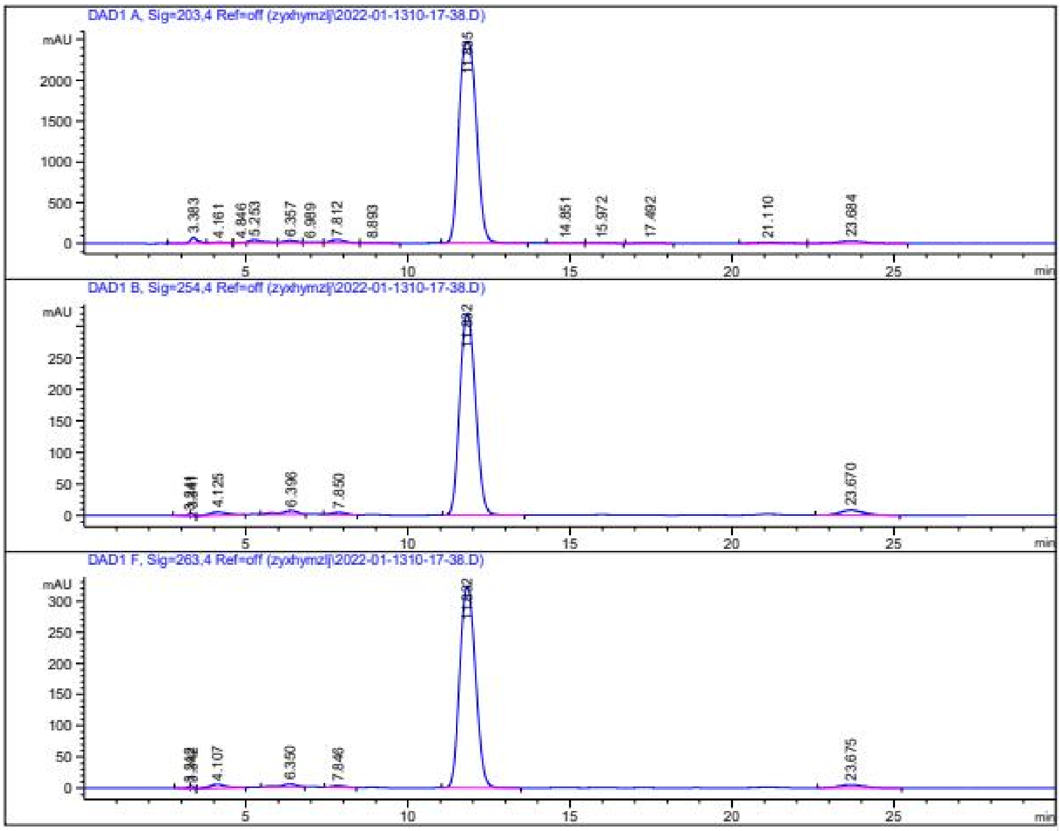

Figure S13. *The HPLC data of* ***4l***

4m:

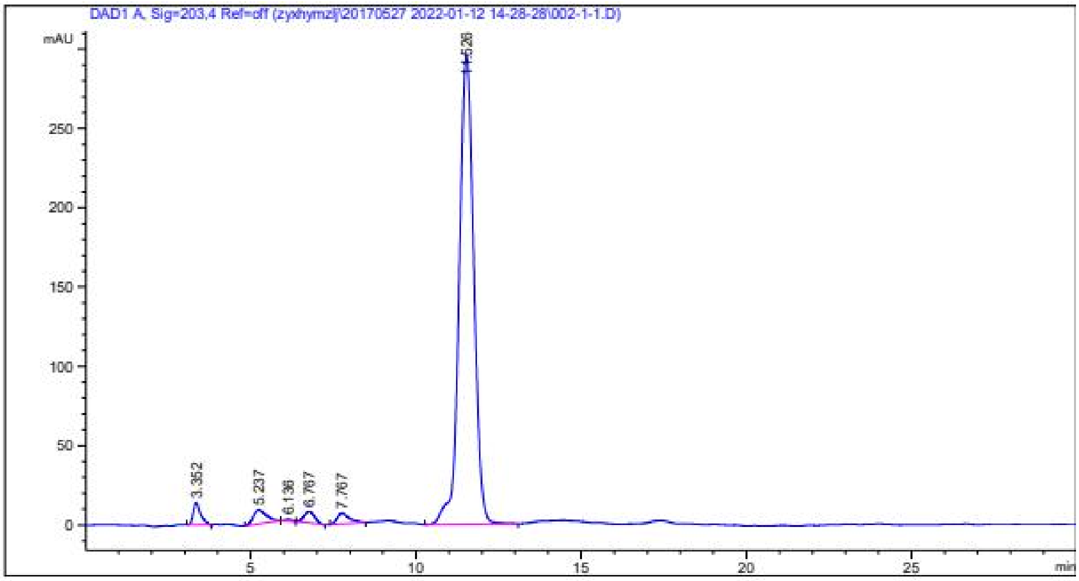

Figure S14. *The HPLC data of* ***4m***

4n:

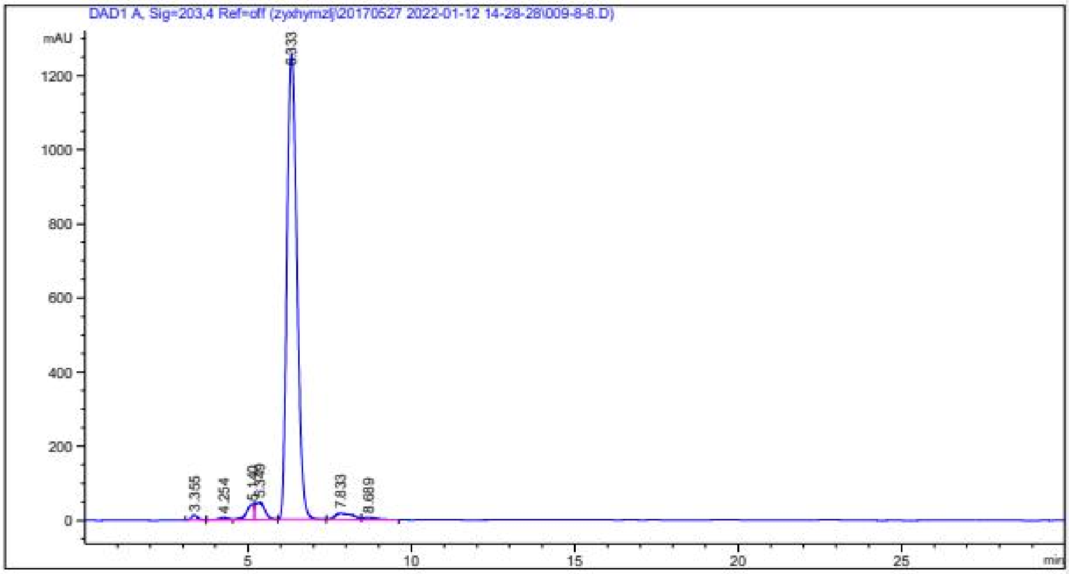

Figure S15. *The HPLC data of* ***4n***

4o:

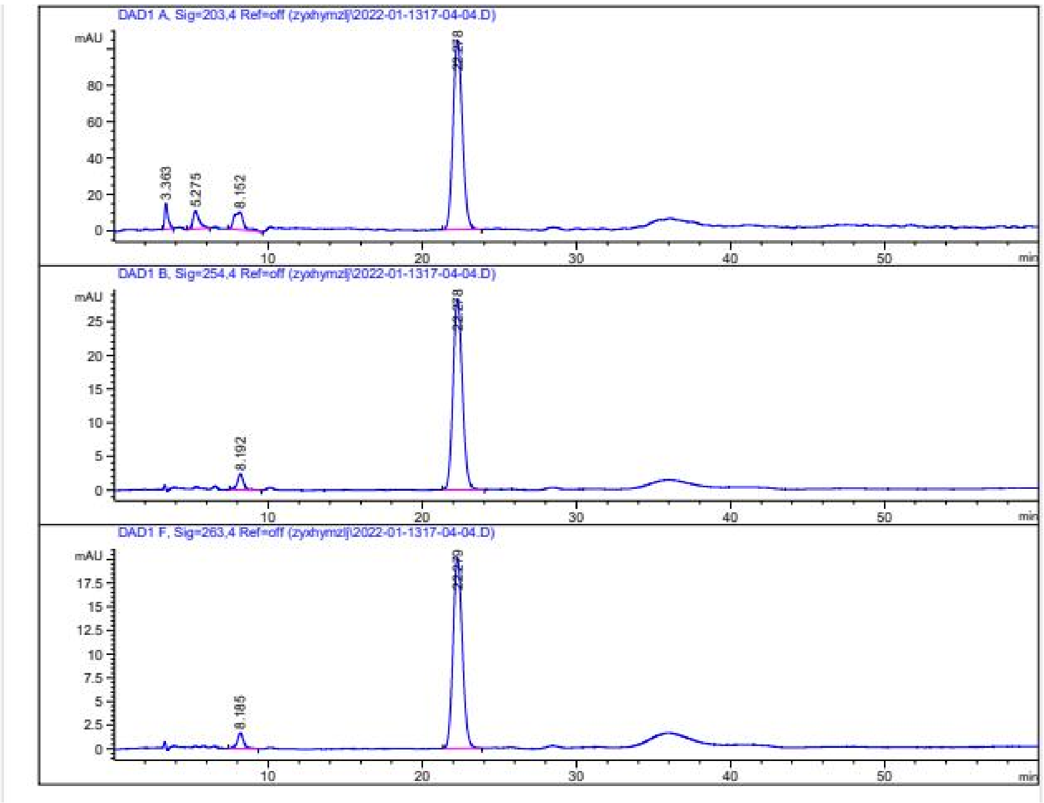

Figure S16. *The HPLC data of* ***4o***

4p:

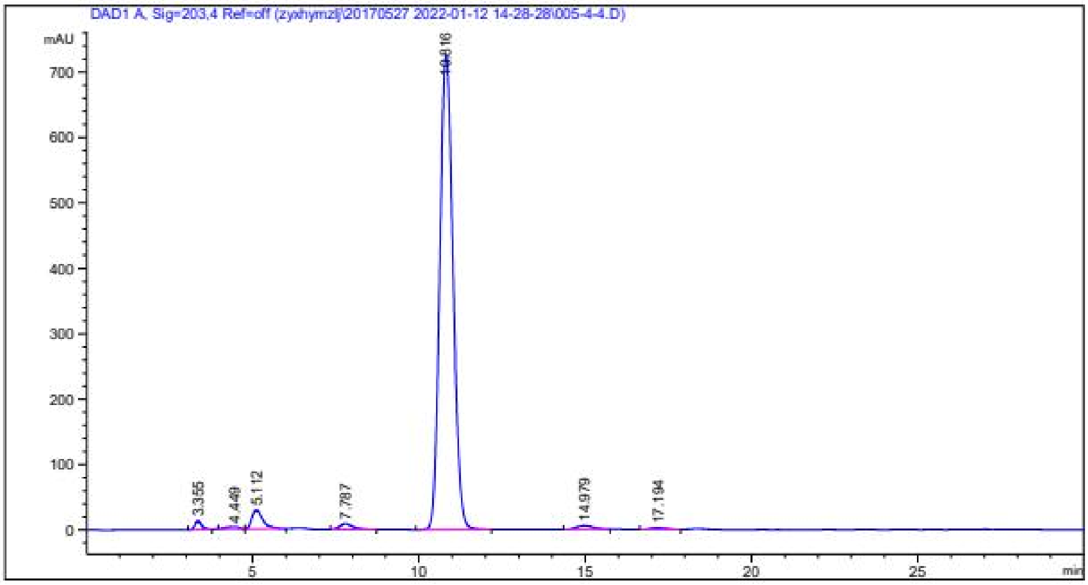

Figure S17. *The HPLC data of* ***4p***

3. NMR Data of Derivatives (**4a-4p**)

4a:

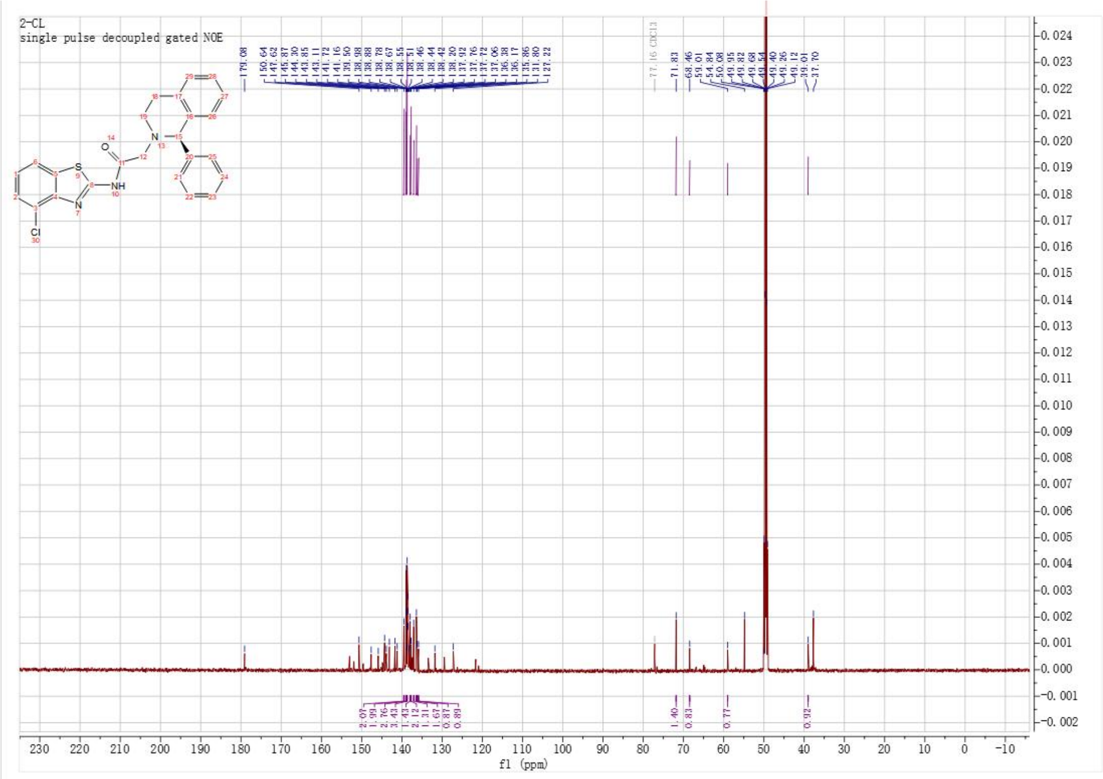

Figure S18. *The* ^{13}*C NMR data of* ***4a***

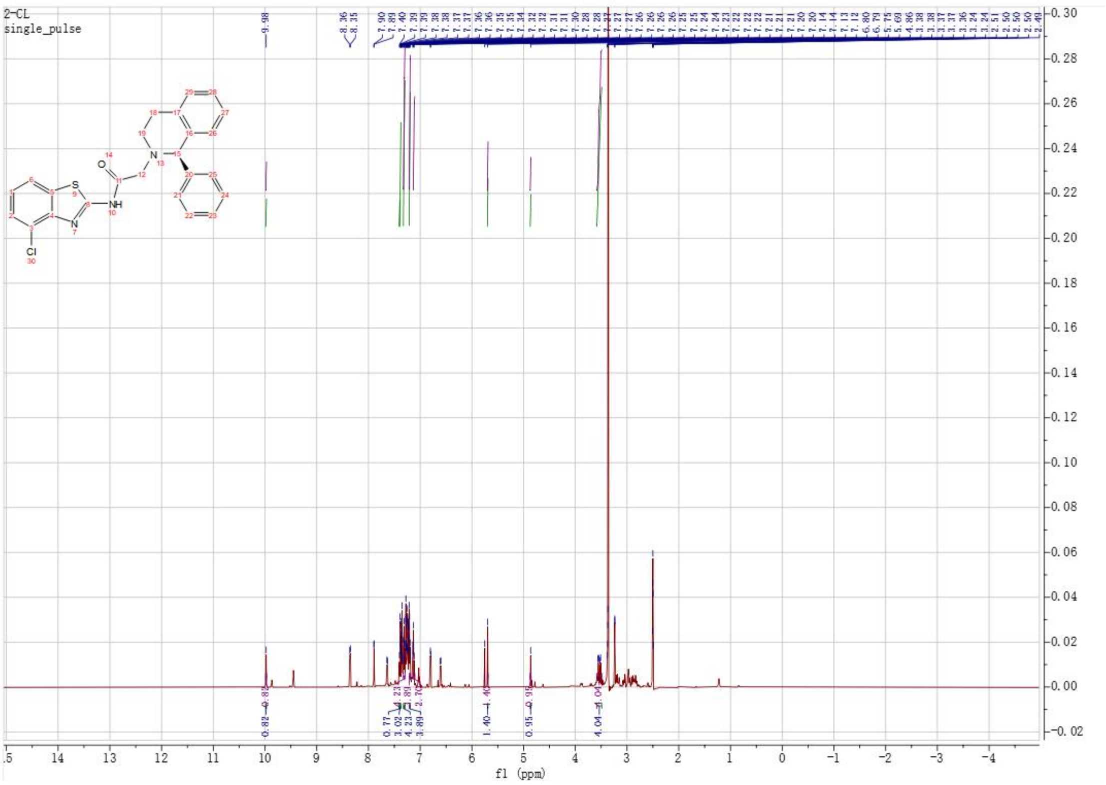

Figure S19. *The* ^{1}*H NMR data of* ***4a***

4b:

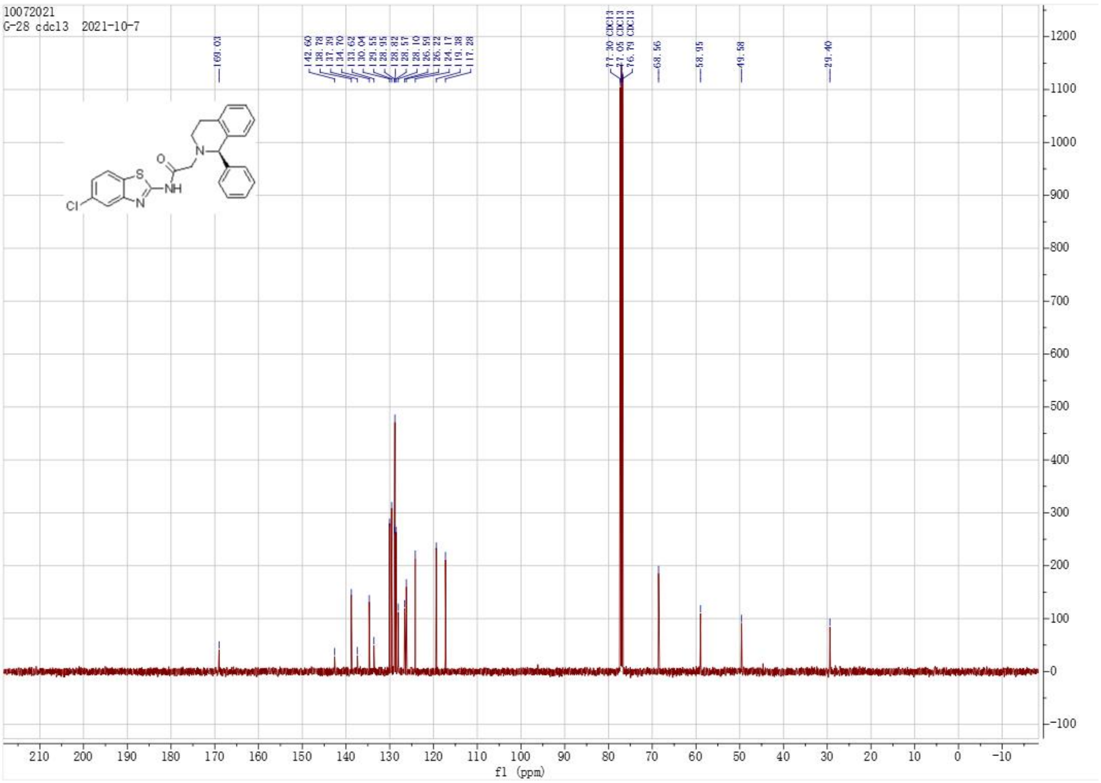

Figure S20. *The* [13]*C NMR data of* ***4b***

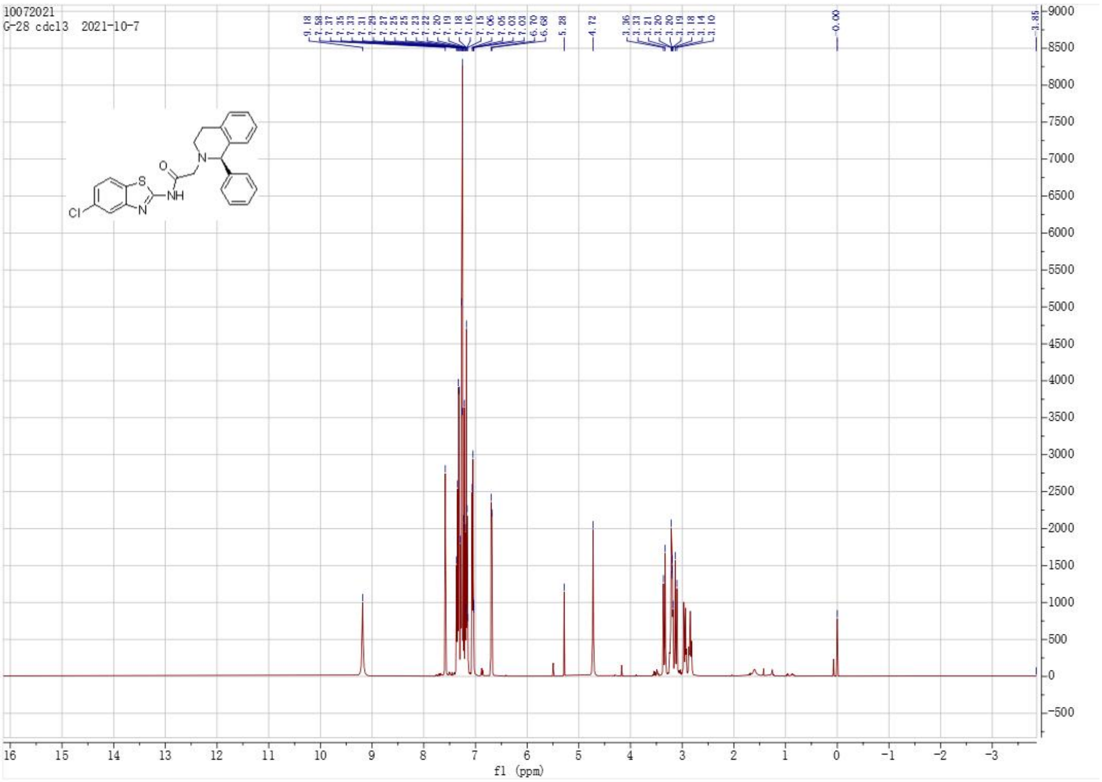

Figure S21. *The* [1]*H NMR data of* ***4b***

4c:

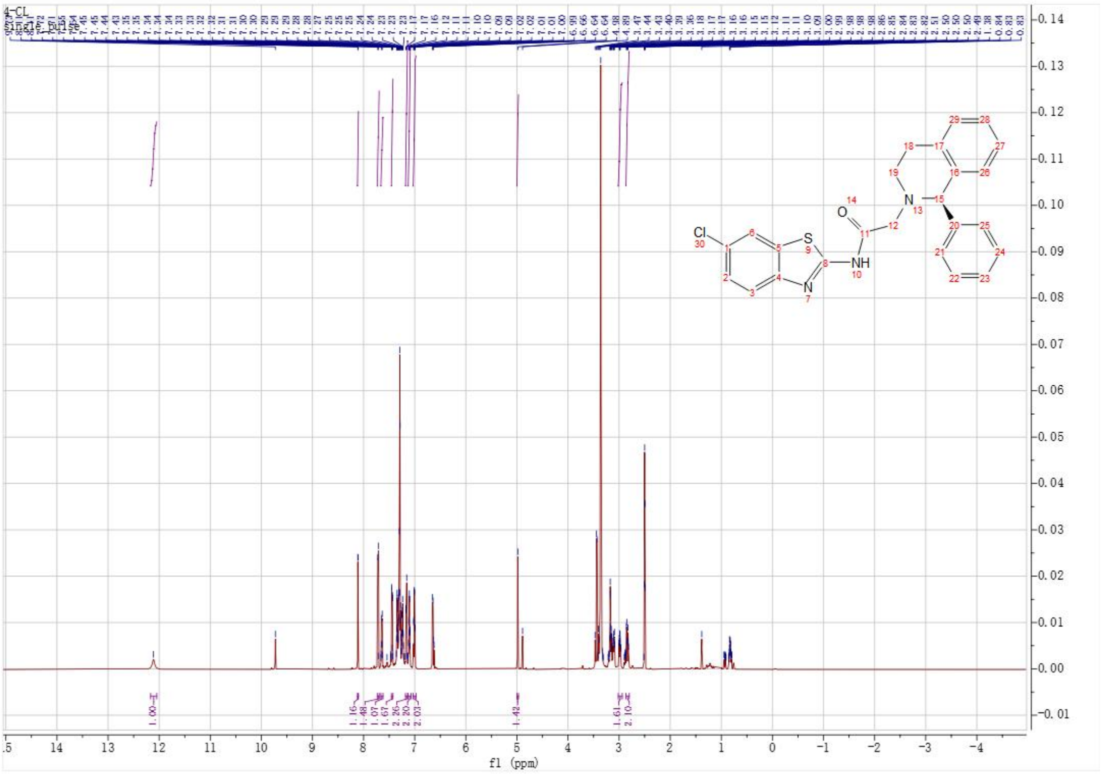

Figure S22. *The ^{13}C NMR data of* ***4c***

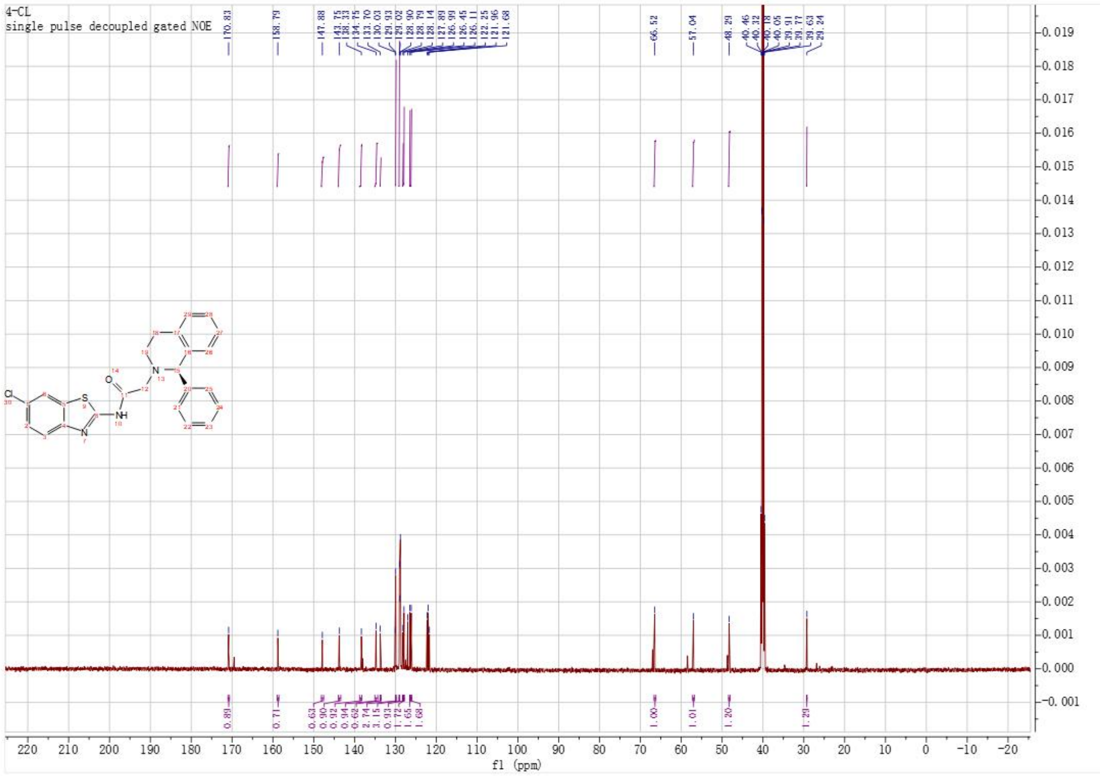

Figure S23. *The ^{1}H NMR data of* ***4c***

4d:

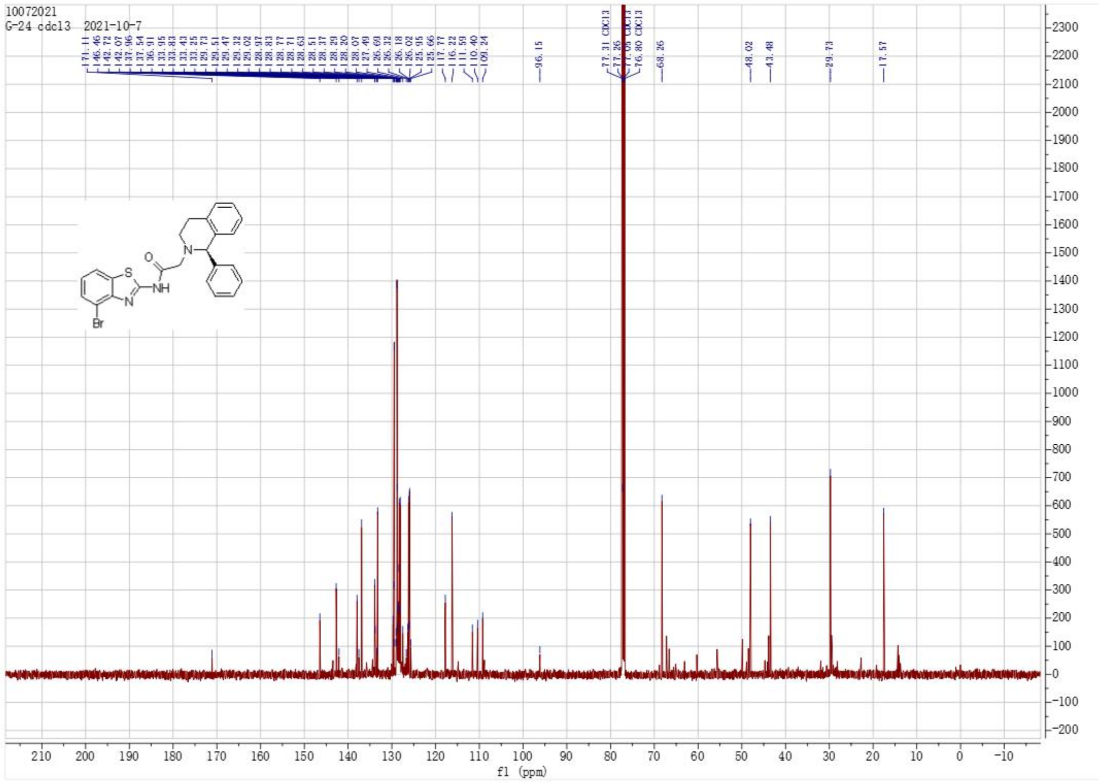

Figure S24. *The* ^{13}C *NMR data of* ***4d***

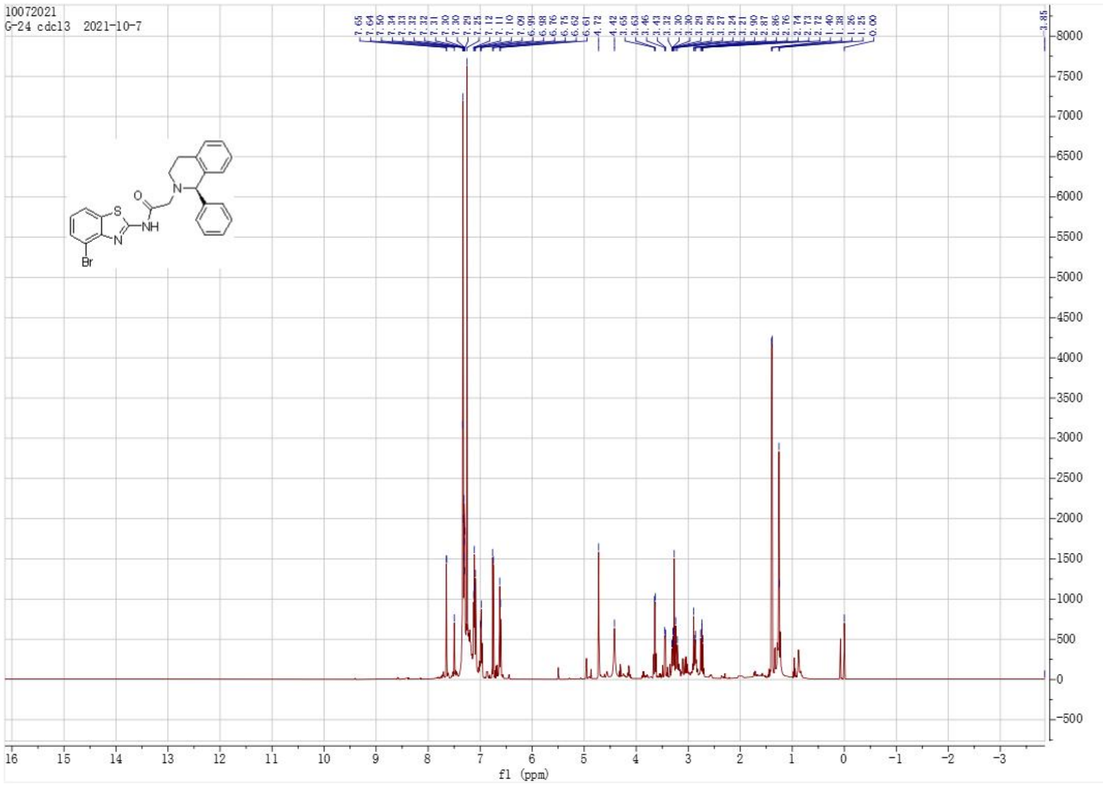

Figure S25. *The* ^{1}H *NMR data of* ***4d***

4e:

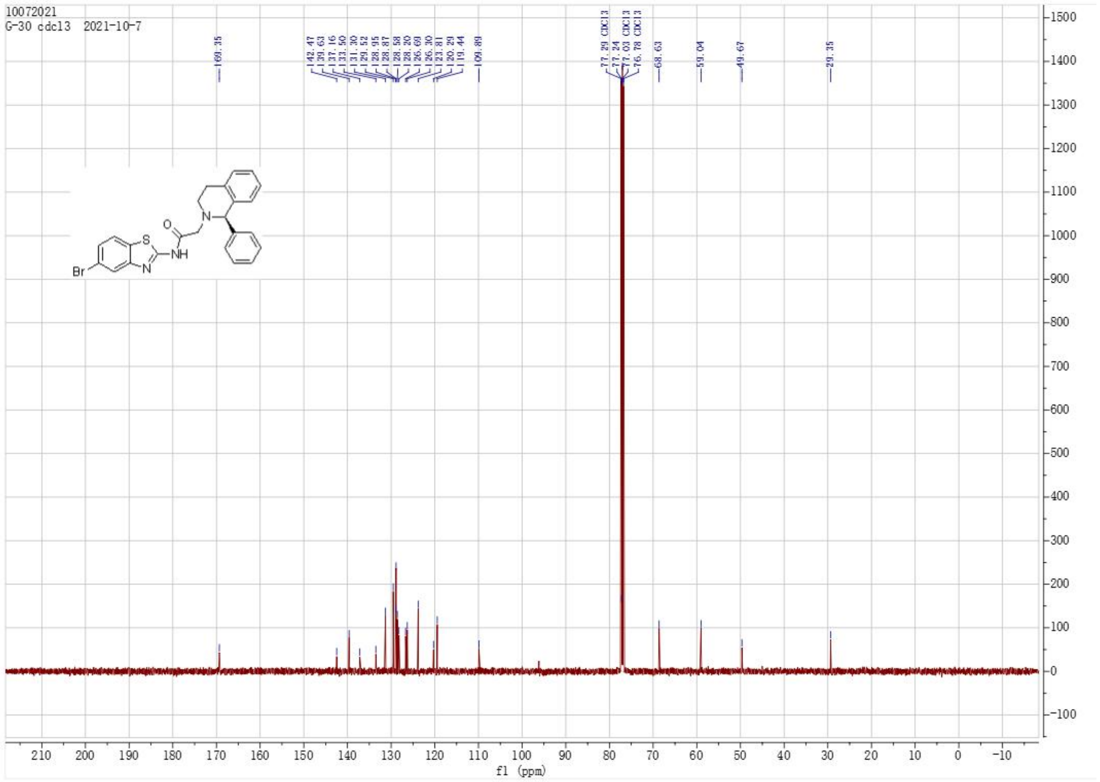

Figure S26. *The* ^{13}C *NMR data of* ***4e***

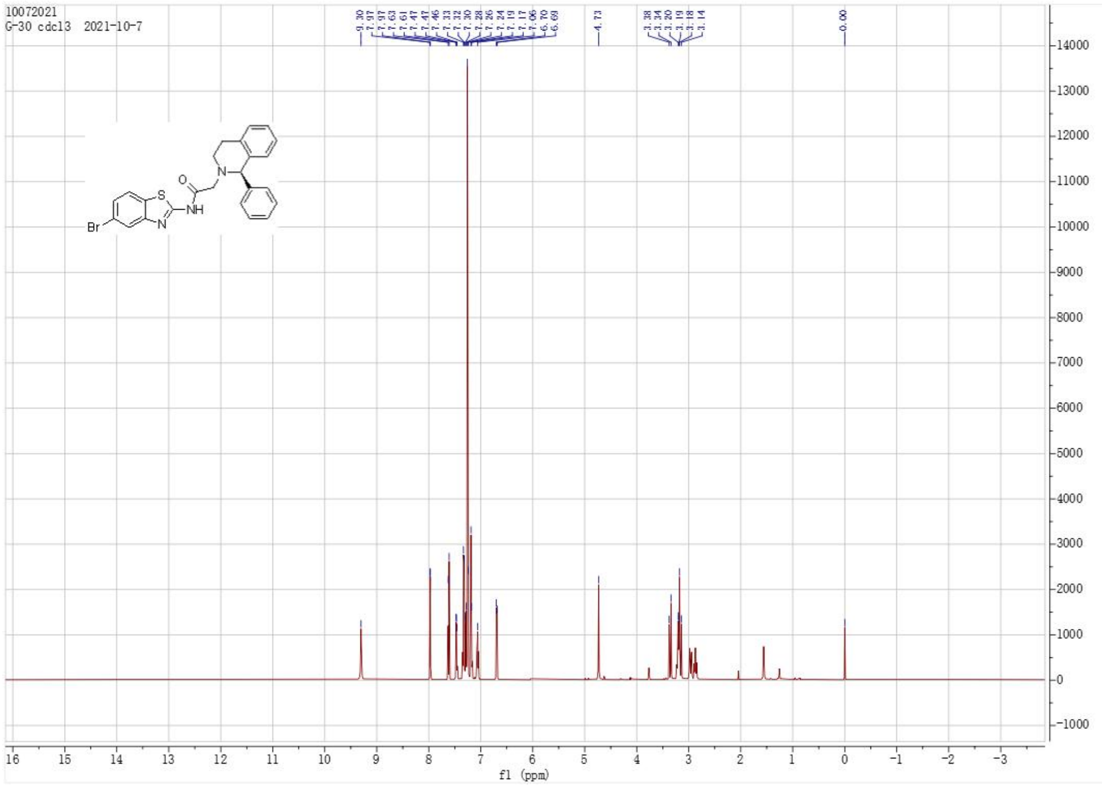

Figure S27. *The* ^{1}H *NMR data of* ***4e***

4f:

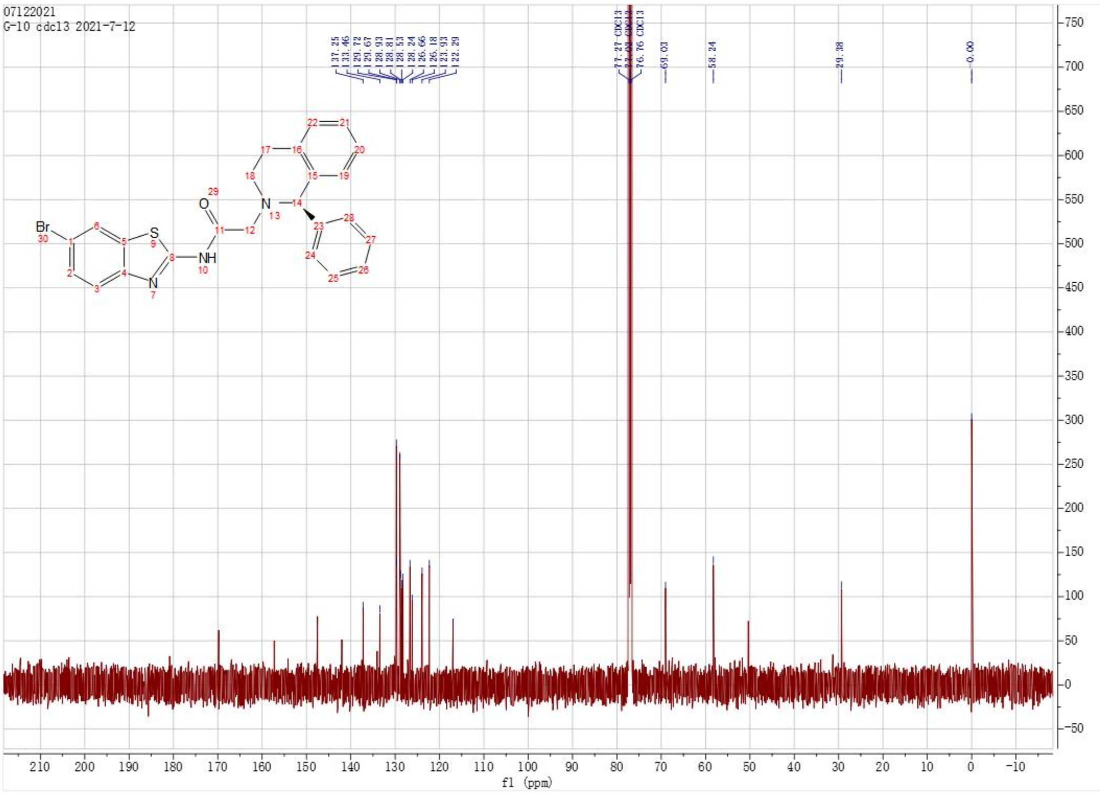

Figure S28. *The* ^{13}*C NMR data of* ***4f***

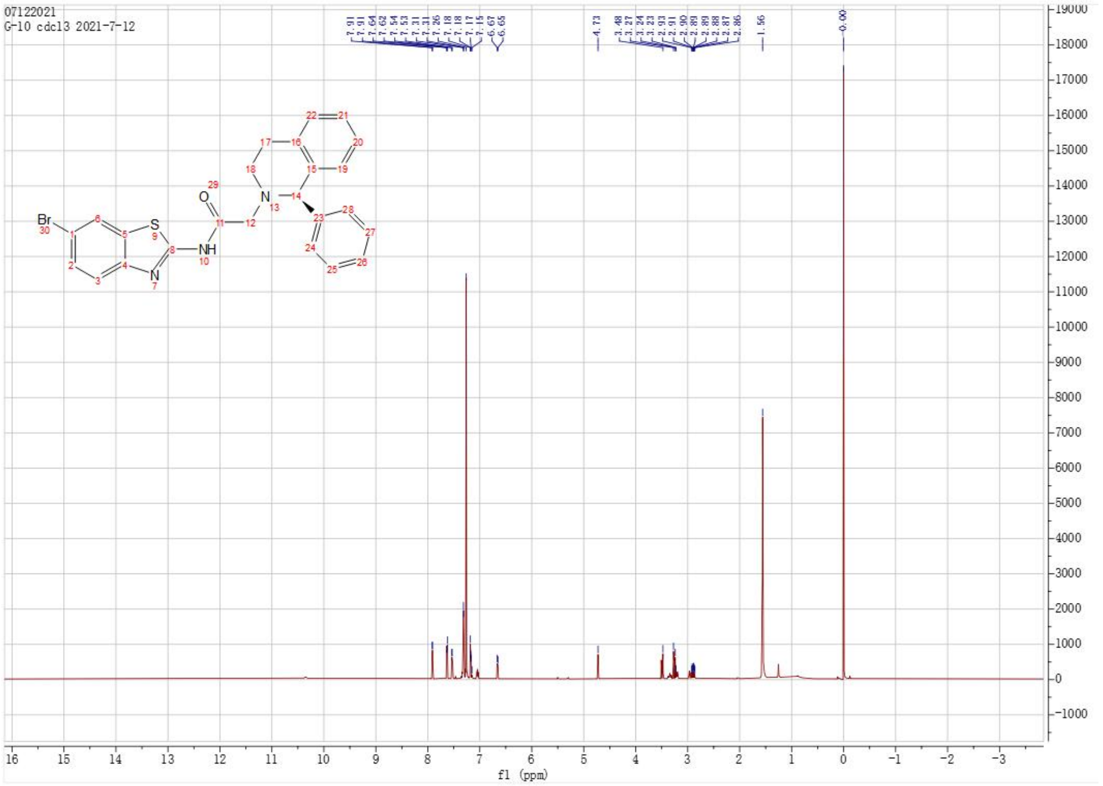

Figure S29. *The* ^{1}*H NMR data of* ***4f***

4g:

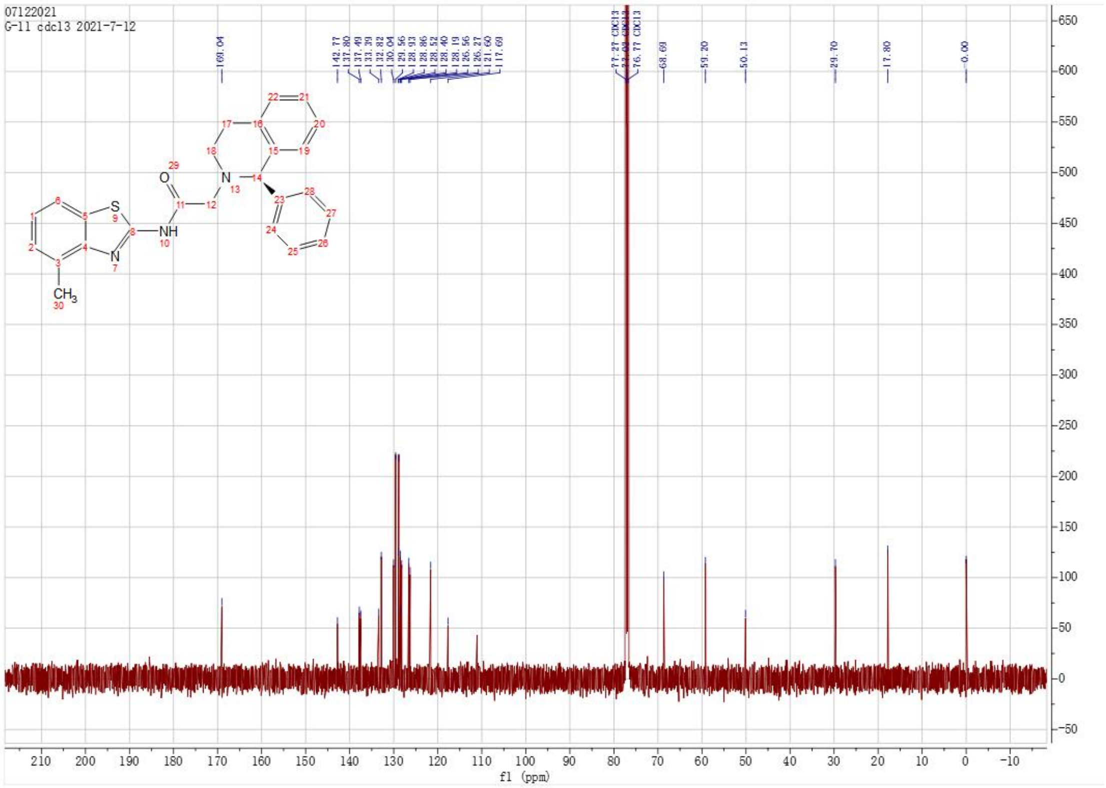

Figure S30. *The* ^{13}C *NMR data of* ***4g***

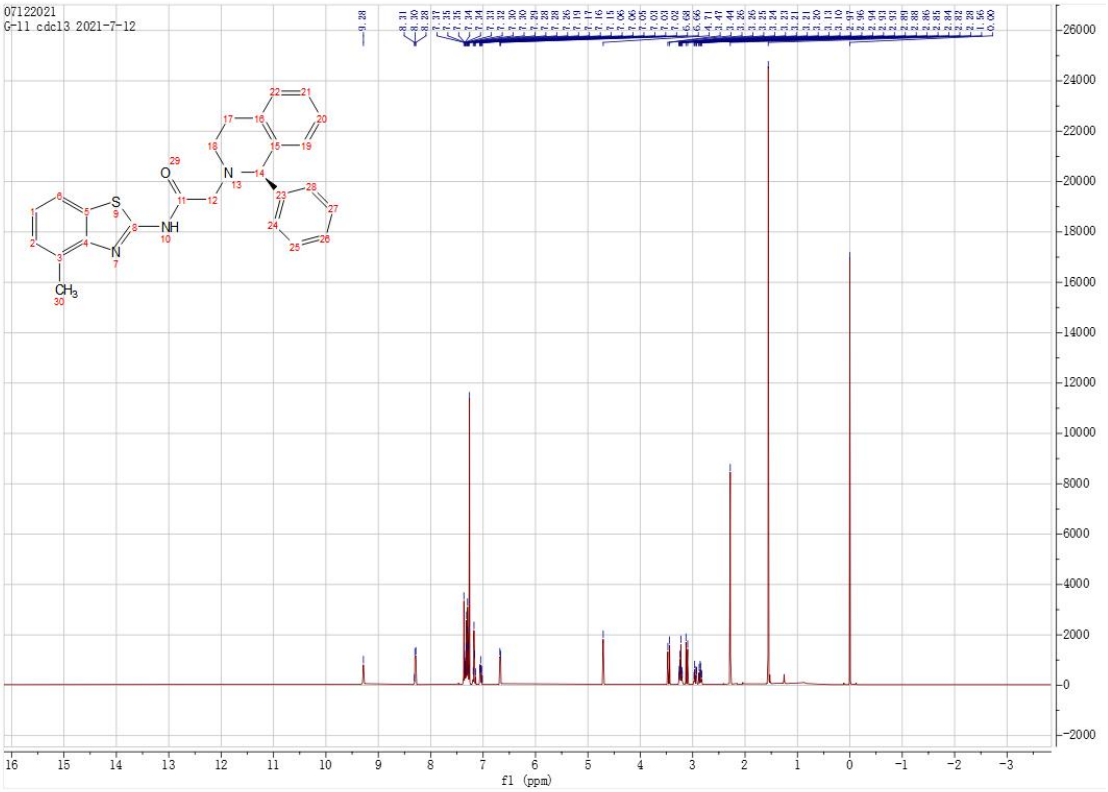

Figure S31. *The* ^{1}H *NMR data of* ***4g***

4h:

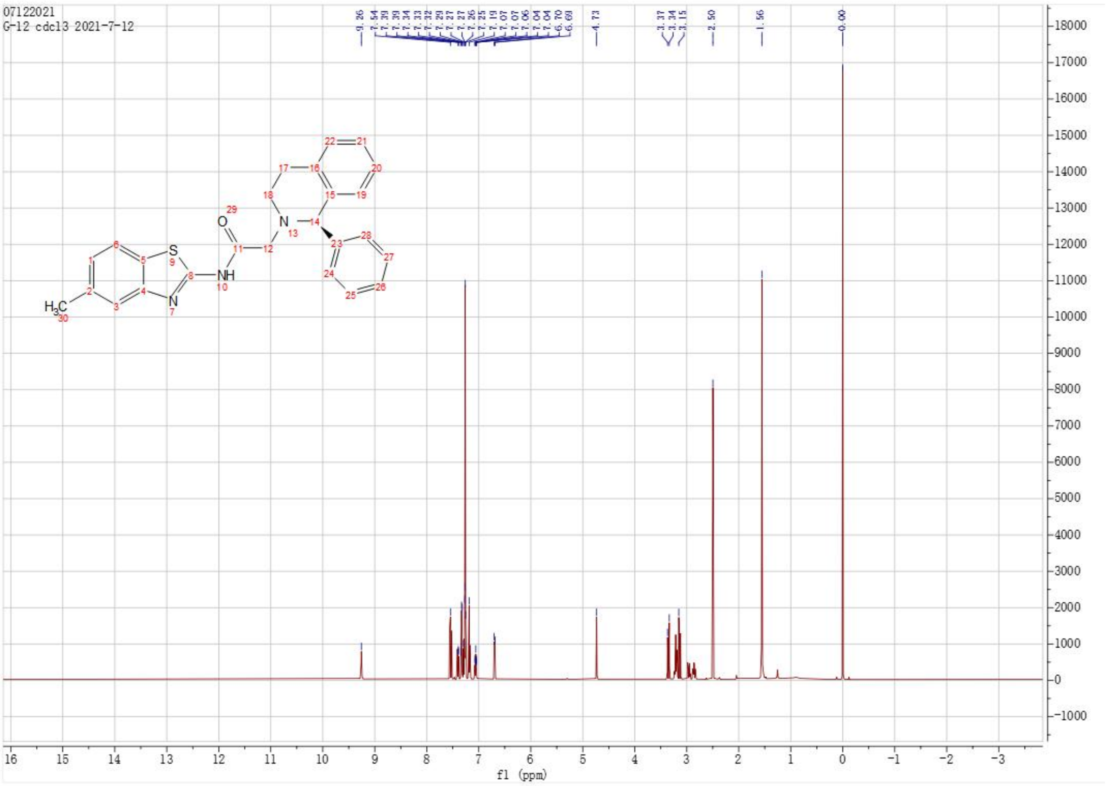

Figure S32. *The* ^{13}*C NMR data of* ***4h***

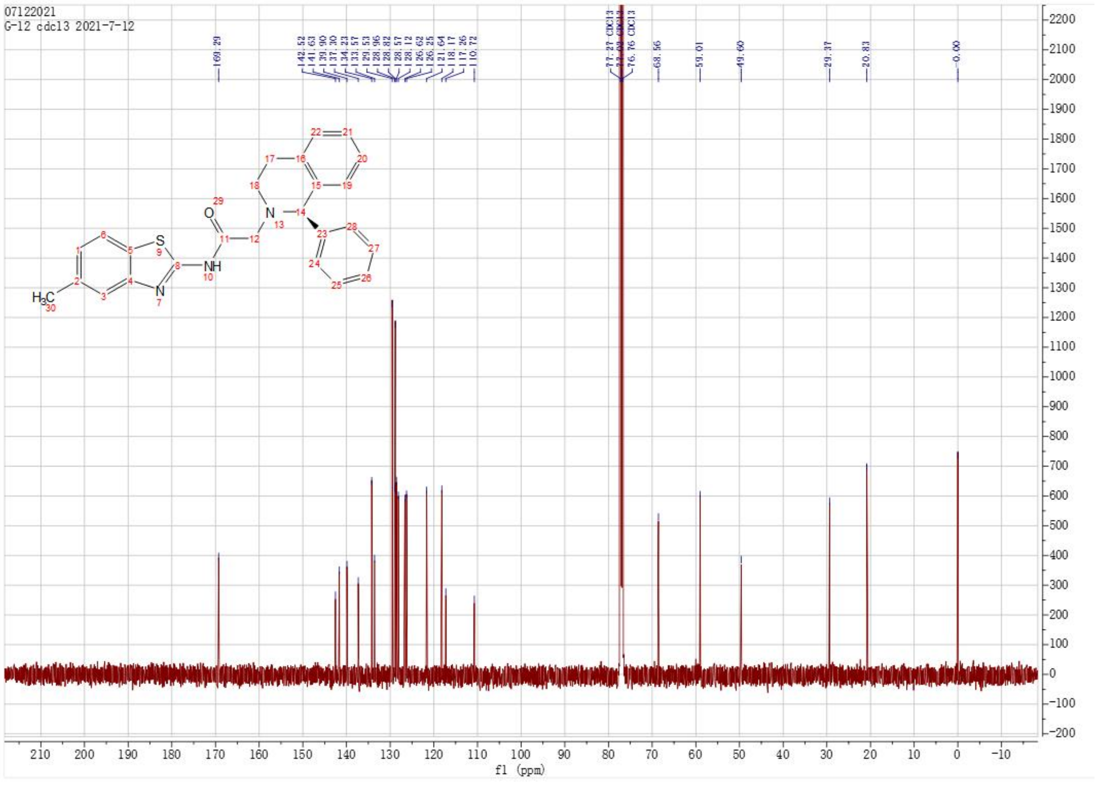

Figure S33. *The* ^{1}*H NMR data of* ***4h***

4i:

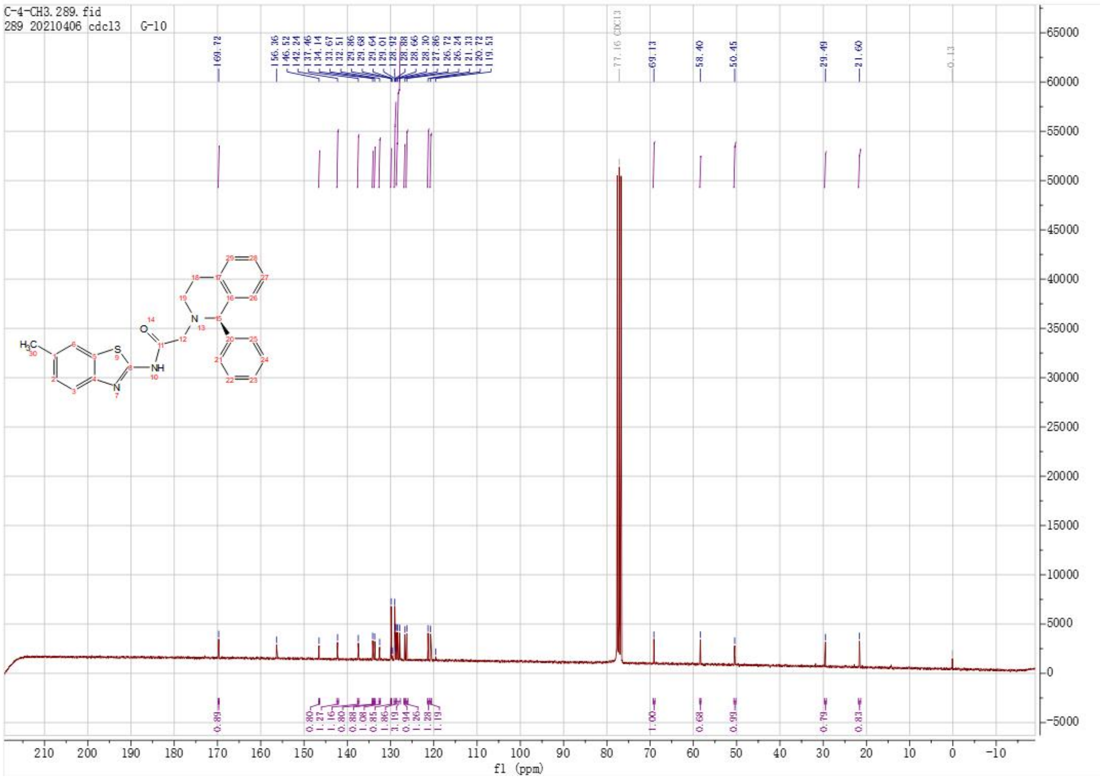

Figure S34. *The* ^{13}*C NMR data of* ***4i***

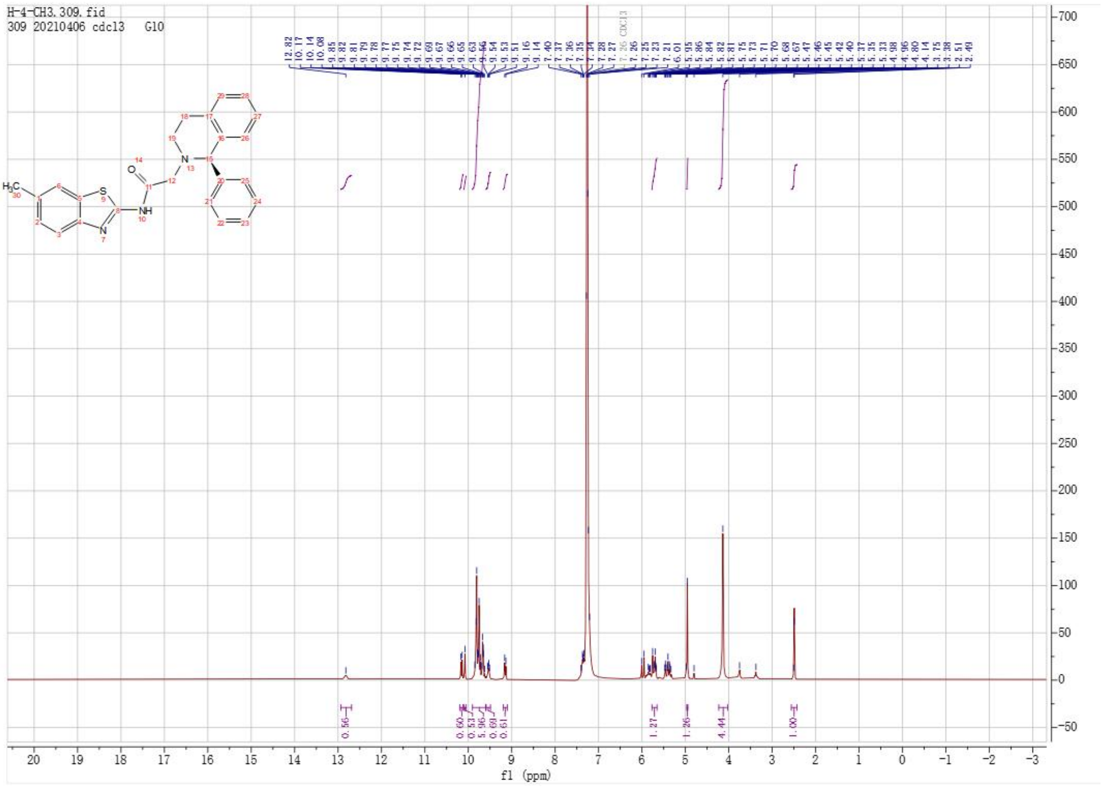

Figure S35. *The* ^{1}*H NMR data of* ***4i***

4j:

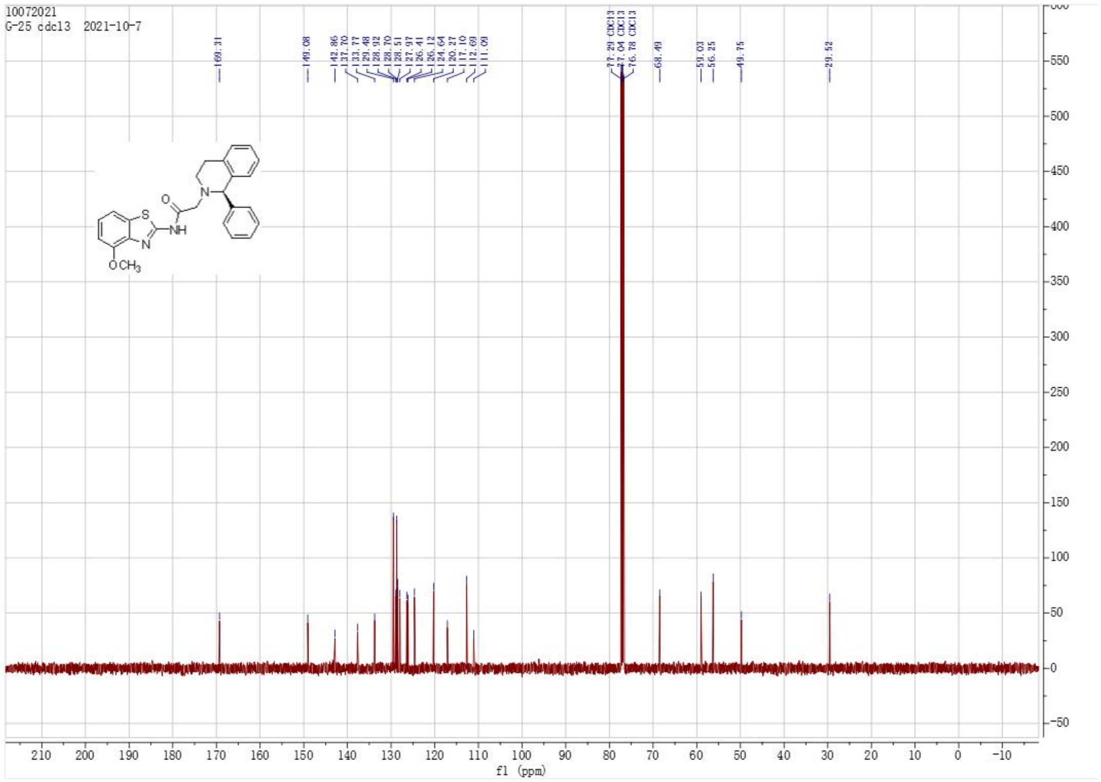

Figure S36. *The* ^{13}C *NMR data of* ***4j***

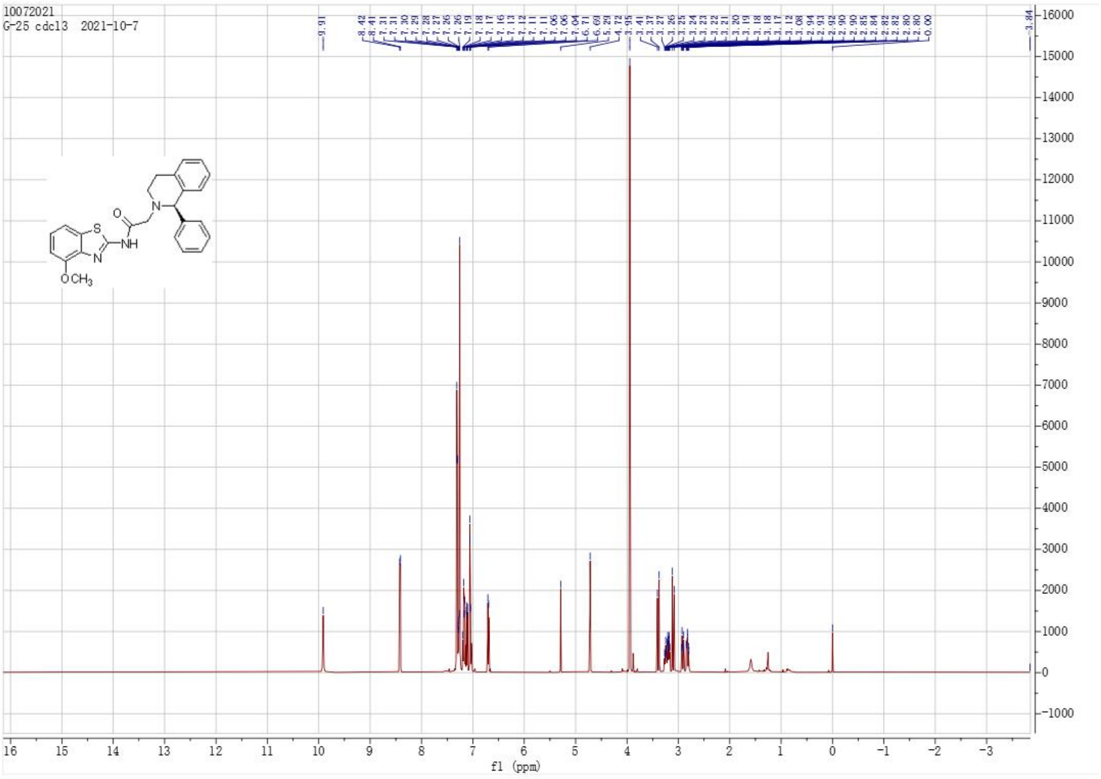

Figure S37. *The* ^{1}H *NMR data of* ***4j***

4k:

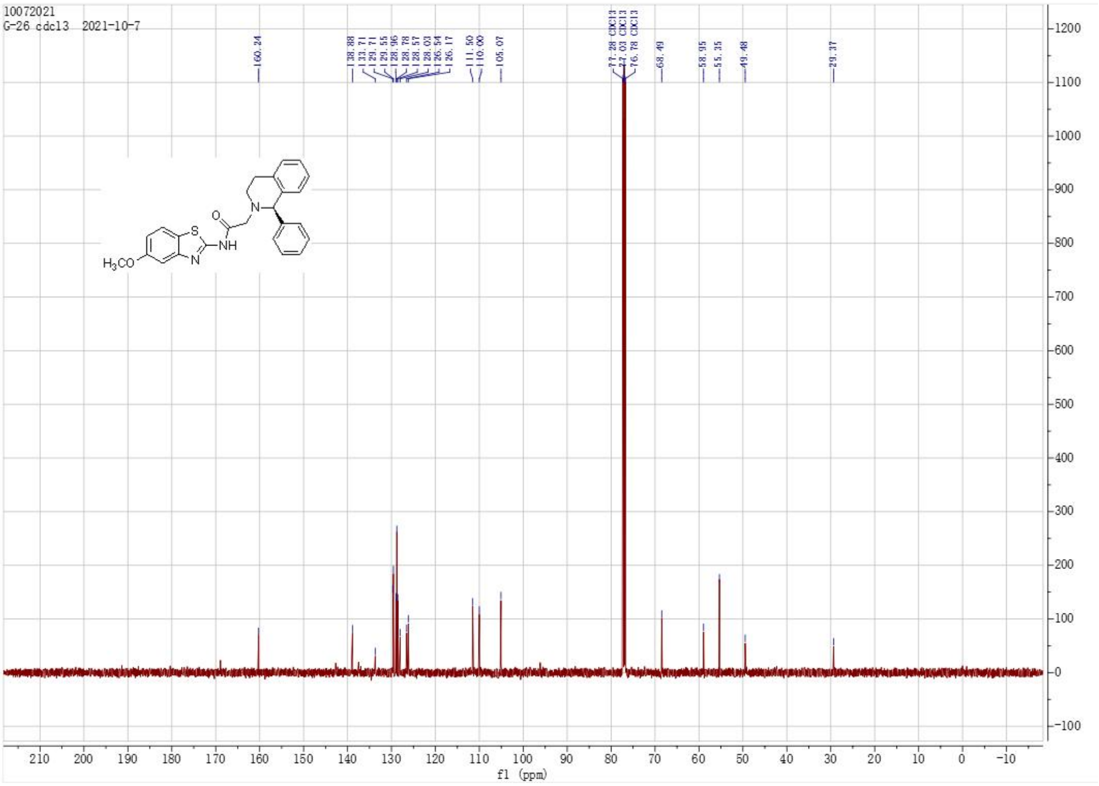

Figure S38. *The ^{13}C NMR data of* ***4k***

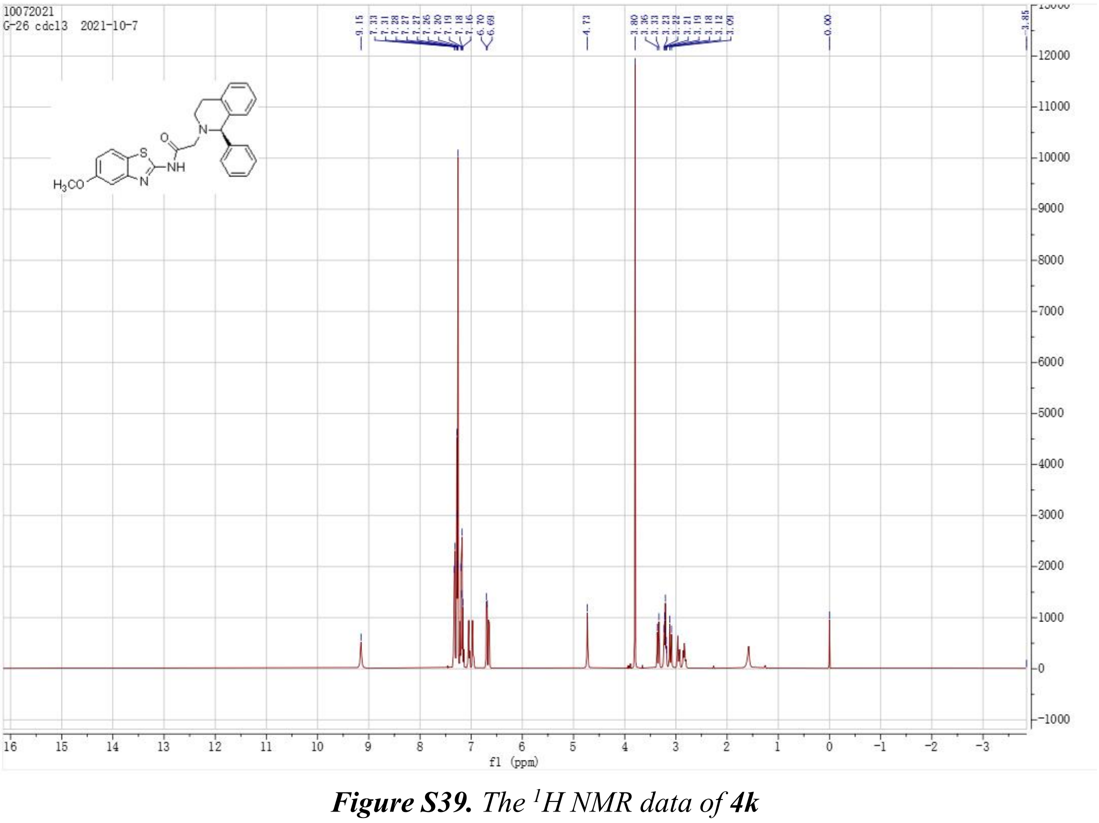

Figure S39. *The ^{1}H NMR data of* ***4k***

4l:

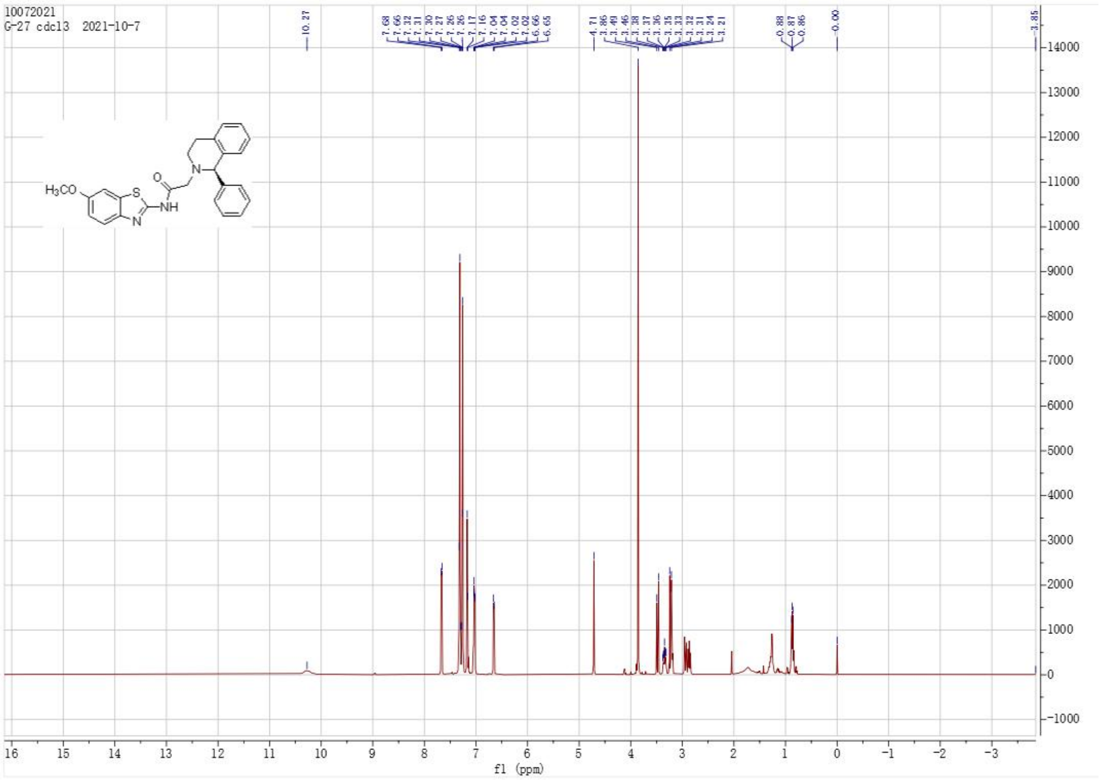

Figure S40. *The ^{13}C NMR data of **4l***

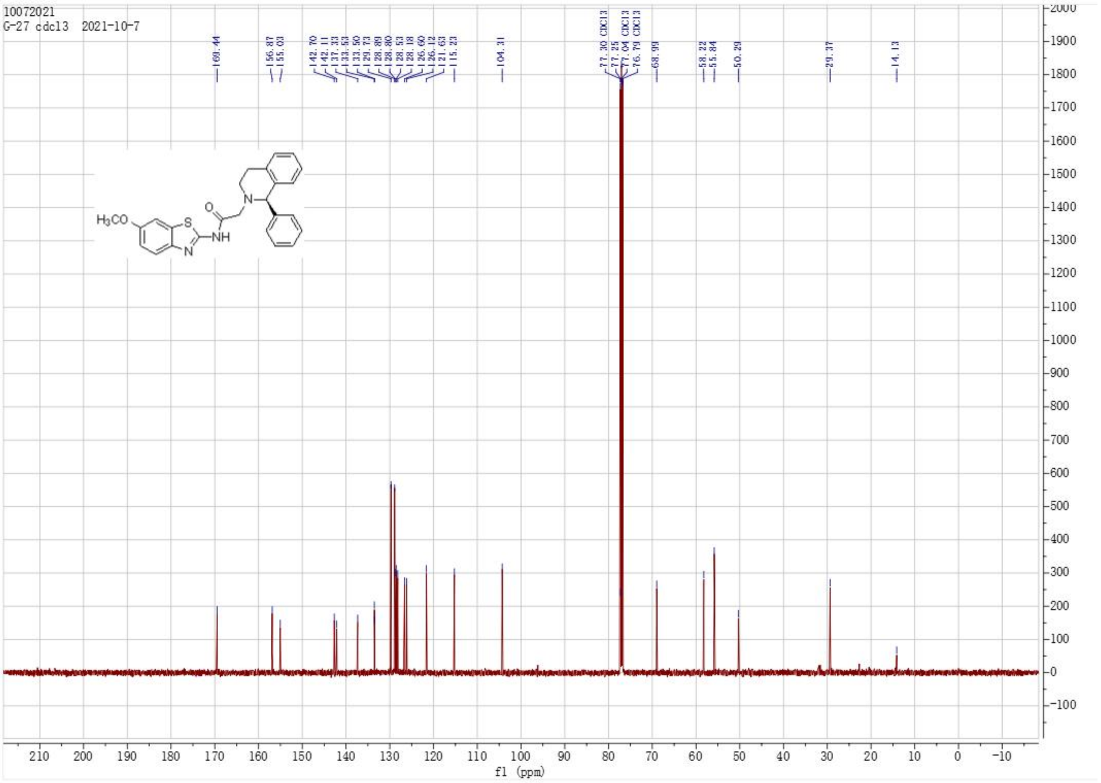

Figure S41. *The ^{1}H NMR data of **4l***

4m:

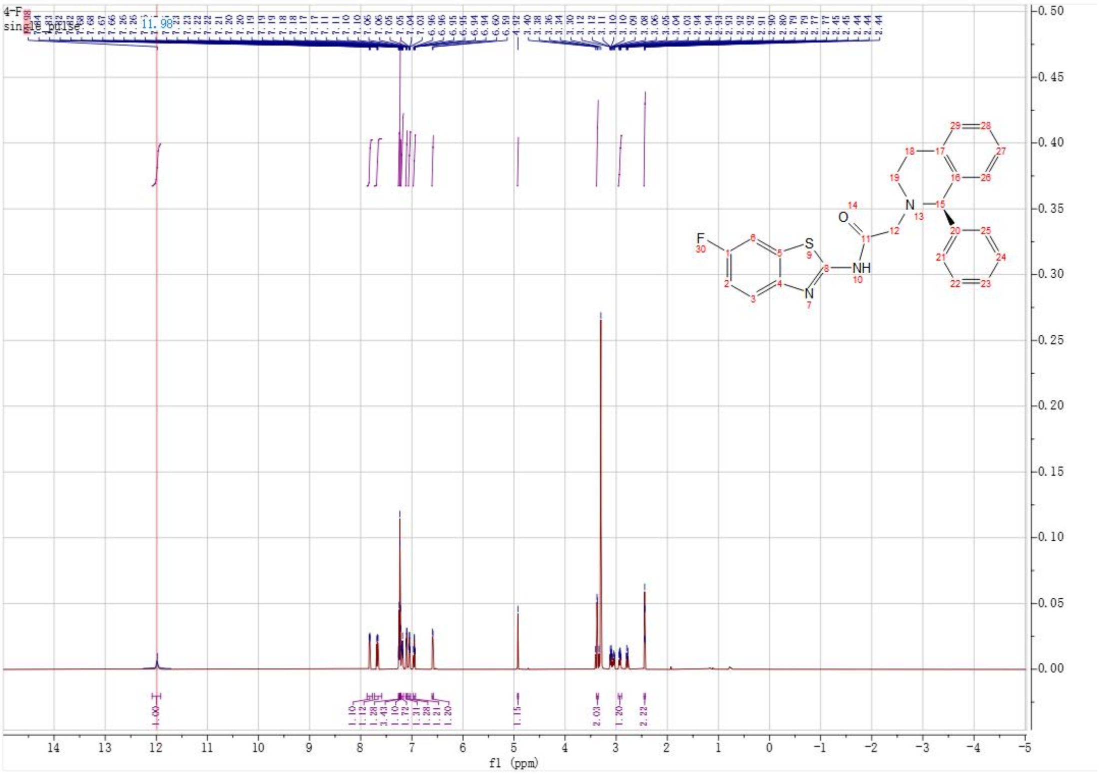

Figure S42. *The ^{13}C NMR data of* ***4m***

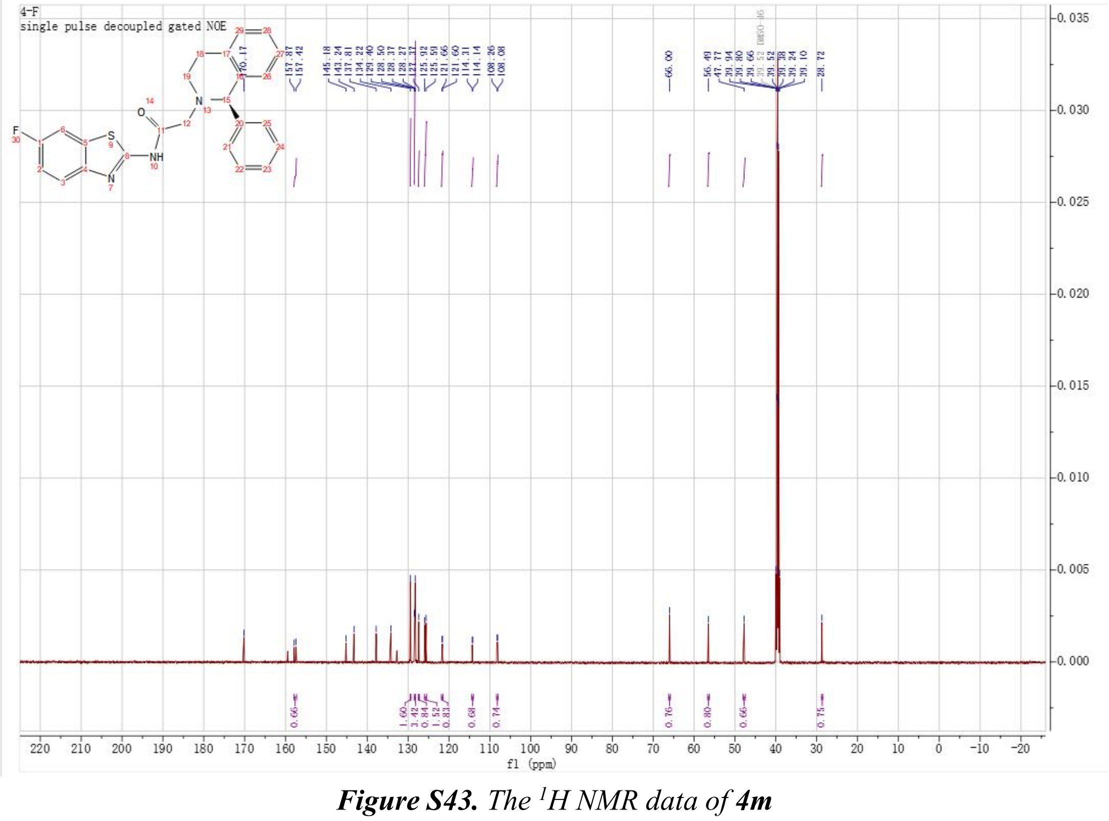

Figure S43. *The ^{1}H NMR data of* ***4m***

4n:

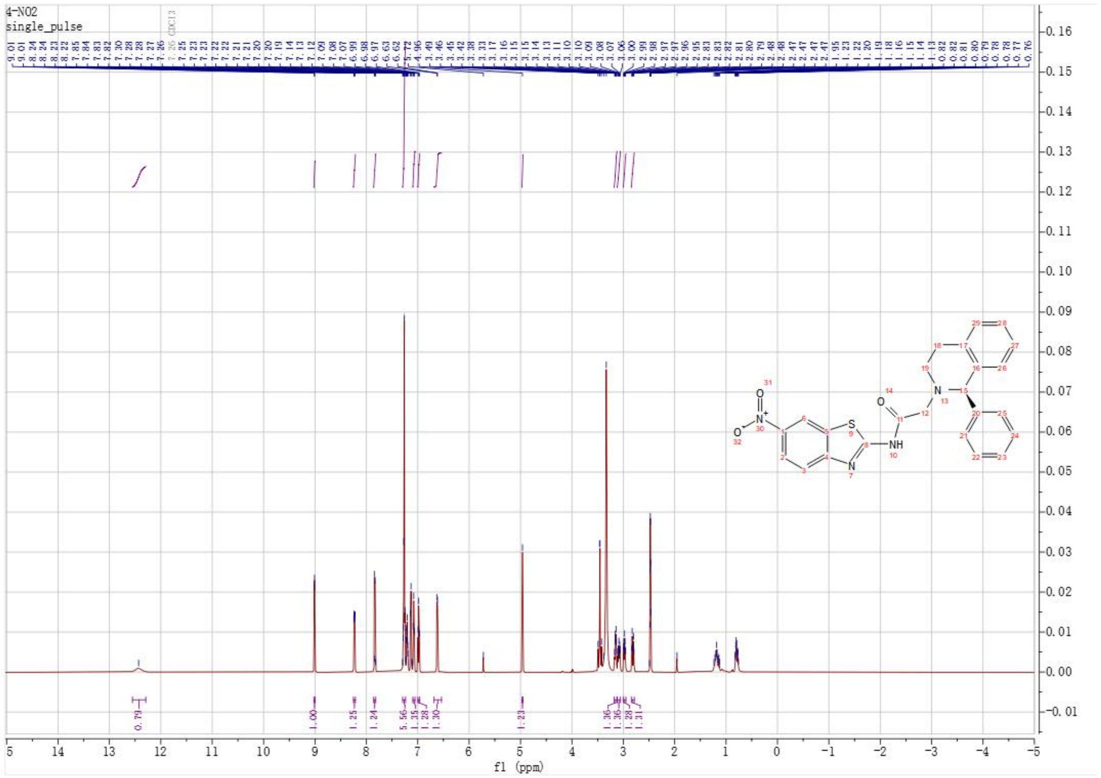

Figure S44. *The* [13]*C NMR data of* ***4n***

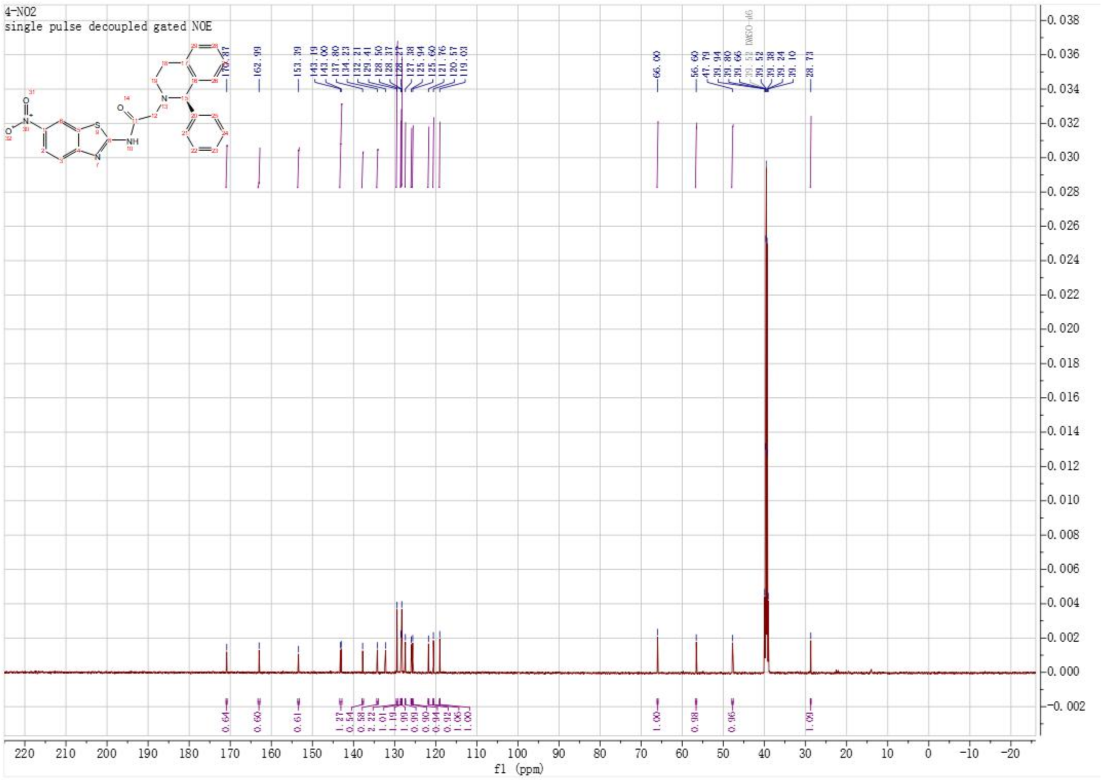

Figure S45. *The* [1]*H NMR data of* ***4n***

4o:

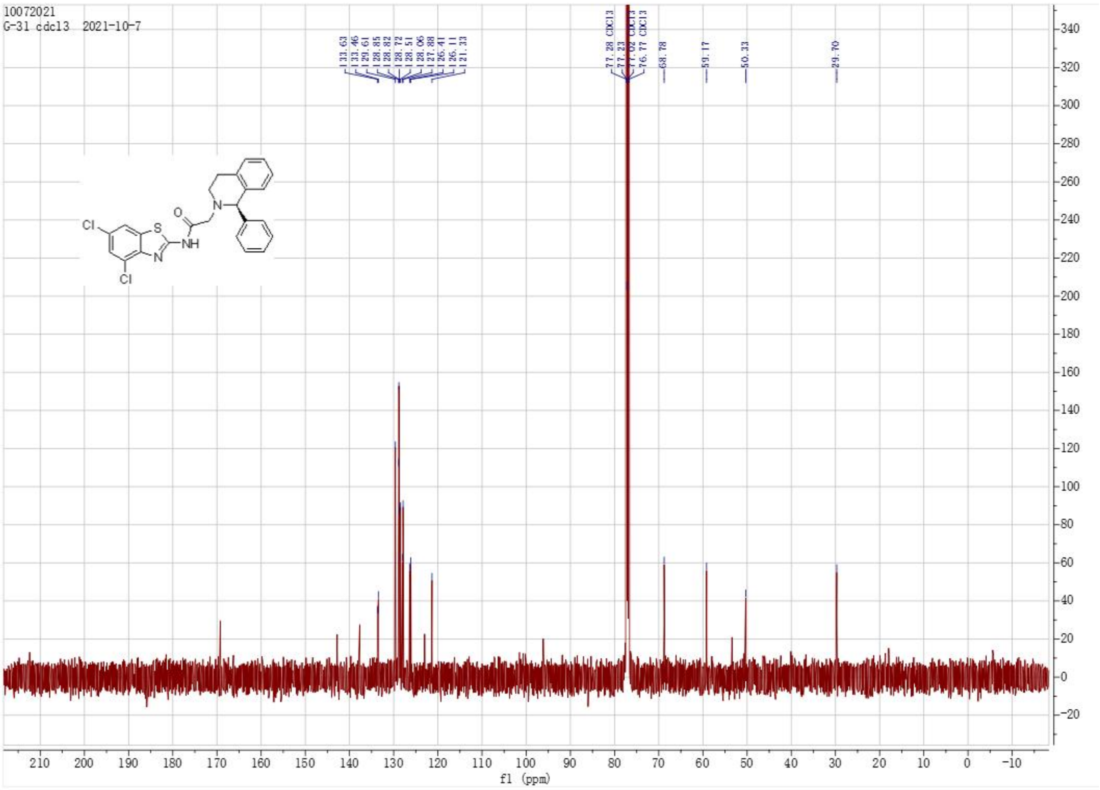

Figure S46. The ^{13}C NMR data of **4o**

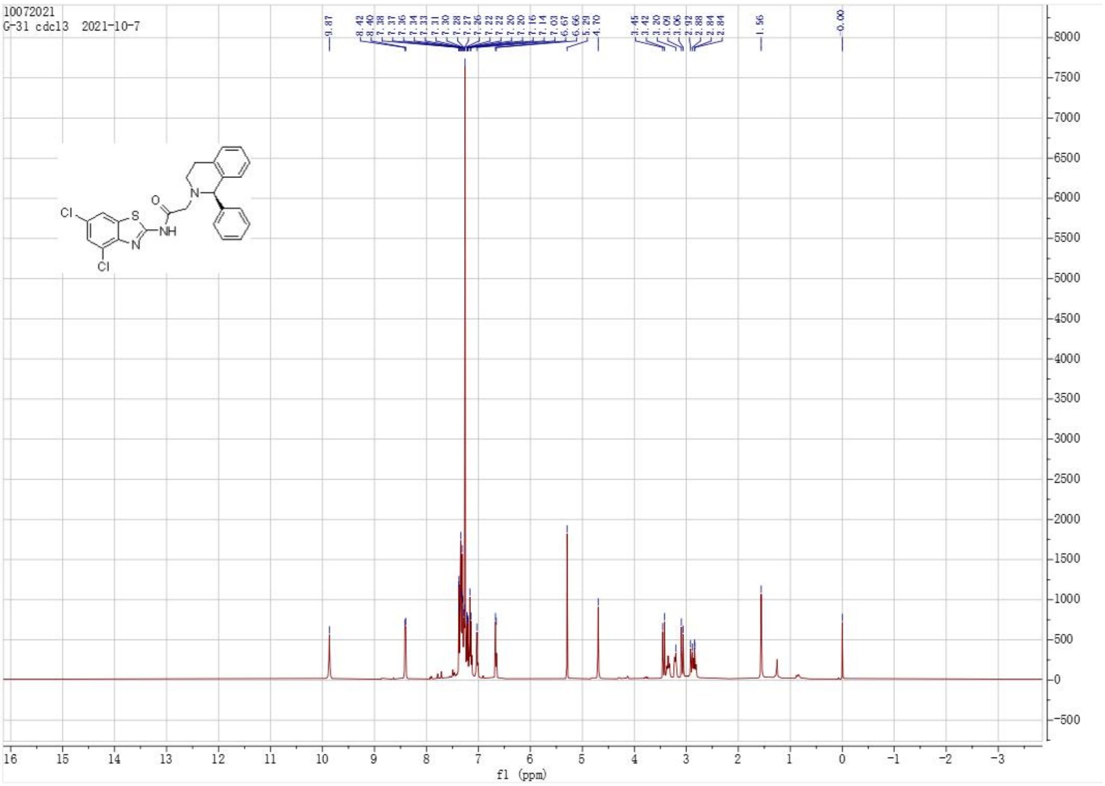

Figure S47. The ^{1}H NMR data of **4o**

4p:

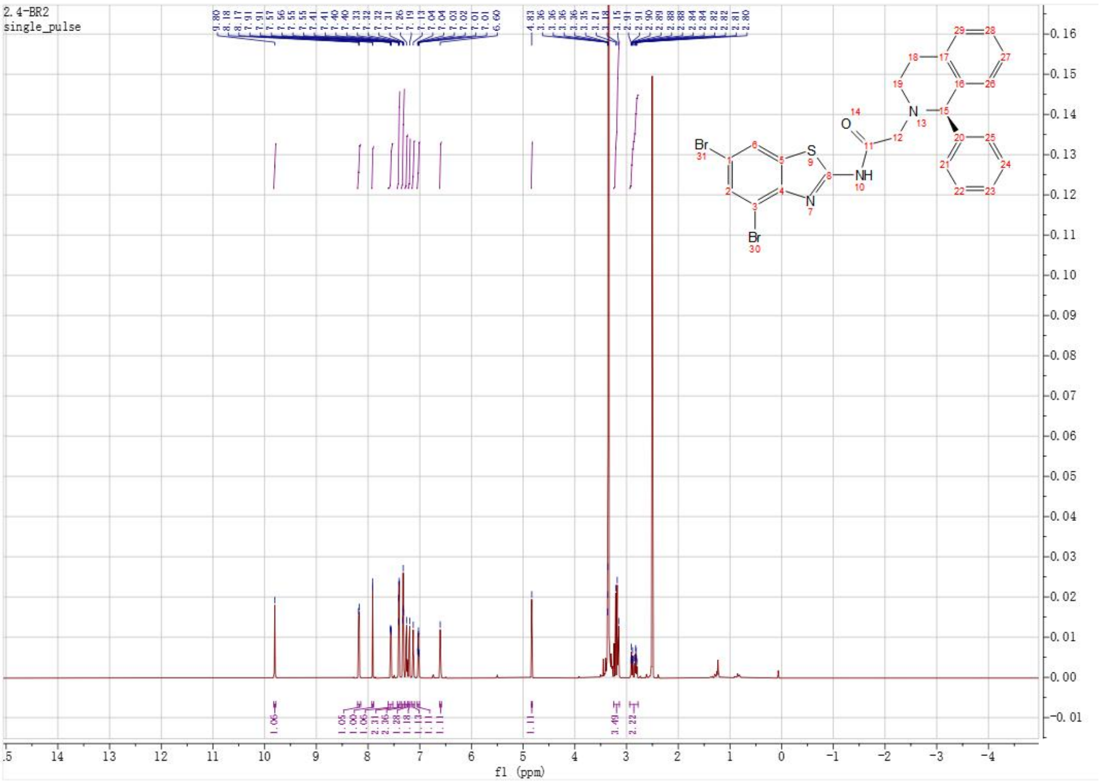

Figure S48. *The ^{13}C NMR data of* ***4p***

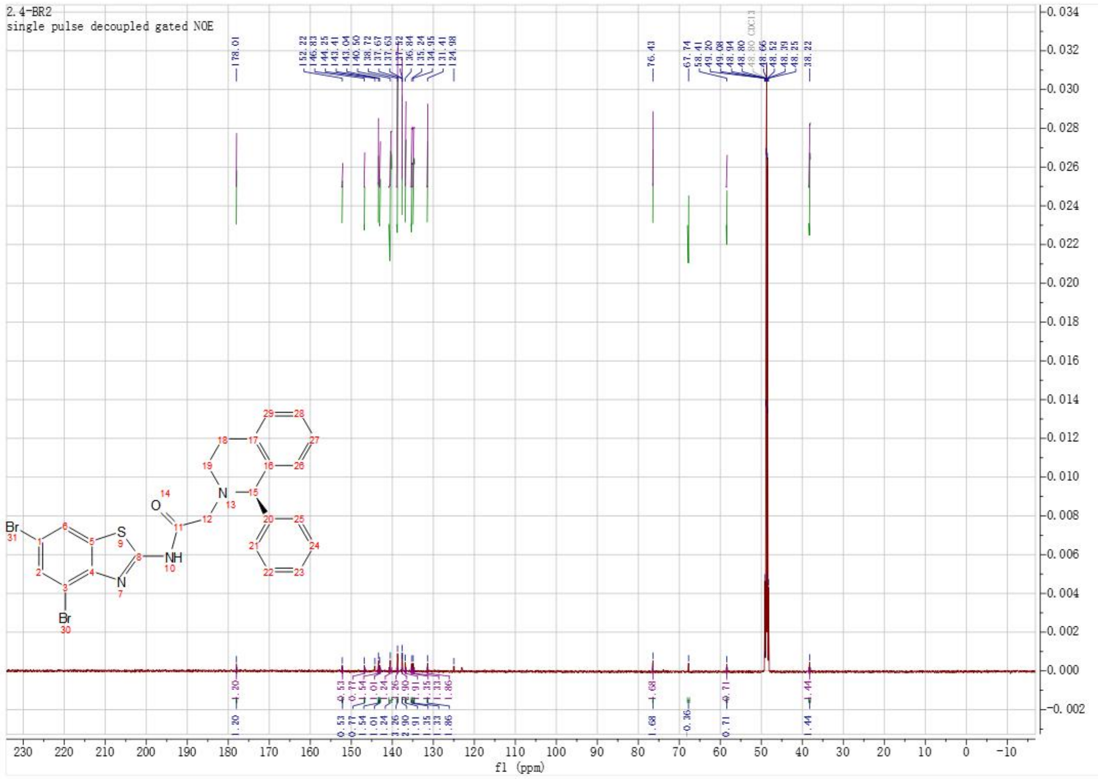

Figure S49. *The ^{1}H NMR data of* ***4p***
